# Chemo-enzymatic synthesis and biological activity evaluation of propenylbenzene derivatives

**DOI:** 10.3389/fmicb.2023.1223123

**Published:** 2023-06-26

**Authors:** Dawid Hernik, Ewa Szczepańska, Maria Chiara Ghezzi, Elisabetta Brenna, Aleksandra Włoch, Hanna Pruchnik, Malwina Mularczyk, Krzysztof Marycz, Teresa Olejniczak, Filip Boratyński

**Affiliations:** ^1^Department of Food Chemistry and Biocatalysis, Wroclaw University of Environmental and Life Sciences, Wrocław, Poland; ^2^Dipartimento di Chimica, Materiali ed Ingegneria Chimica “Giulio Natta”, Politecnico di Milano, Milan, Italy; ^3^Department of Physics and Biophysics, Wroclaw University of Environmental and Life Sciences, Wrocław, Poland; ^4^Department of Experimental Biology, Wroclaw University of Environmental and Life Sciences, Wrocław, Poland

**Keywords:** biotransformation, fragrances, propenylbenzenes, oxidation, fungistatic activity, antioxidant activity, haemolytic activity, proliferative activity

## Abstract

Propenylbenzenes, including isosafrole, anethole, isoeugenol, and their derivatives, are natural compounds found in essential oils from various plants. Compounds of this group are important and valuable, and are used in the flavour and fragrance industries as well as the pharmaceutical and cosmetic industries. The aim of this study was to develop an efficient process for synthesising oxygenated derivatives of these compounds and evaluate their potential biological activities. In this paper, we propose a two-step chemo-enzymatic method. The first step involves the synthesis of corresponding diols **1b–5b** from propenylbenzenes **1a–5a***via* lipase catalysed epoxidation followed by epoxide hydrolysis. The second step involves the microbial oxidation of a diasteroisomeric mixture of diols **1b–5b** to yield the corresponding hydroxy ketones **1c–4c**, which in this study was performed on a preparative scale using *Dietzia* sp. DSM44016, *Rhodococcus erythropolis* DSM44534, *R. erythropolis* PCM2150, and *Rhodococcus ruber* PCM2166. Application of scaled-up processes allowed to obtain hydroxy ketones **1-4c** with the following yield range 36–62.5%. The propenylbenzene derivatives thus obtained and the starting compounds were tested for various biological activities, including antimicrobial, antioxidant, haemolytic, and anticancer activities, and their impact on membrane fluidity. Fungistatic activity assay against selected strains of *Candida albicans* results in MIC_50_ value varied from 37 to 124 μg/mL for compounds **1a**, **3a–c**, **4a,b**, and **5a,b**. The highest antiradical activity was shown by propenylbenzenes **1-5a** with a double bond in their structure with EC_50_ value ranged from 19 to 31 μg/mL. Haemolytic activity assay showed no cytotoxicity of the tested compounds on human RBCs whereas, compounds **2b–4b** and **2c–4c** affected the fluidity of the RBCs membrane. The tested compounds depending on their concentration showed different antiproliferative activity against HepG2, Caco-2, and MG63. The results indicate the potential utility of these compounds as fungistatics, antioxidants, and proliferation inhibitors of selected cell lines.

## 1. Introduction

Propenylbenzenes, such as isosafrole, anethol, isoeugenol, and their derivatives, are widely found in essential oils from plants such as the aniseed tree, liquorice, and the cananga tree (Newberne et al., 1999; Thi Luu et al., 2009; Fahlbusch et al., 2003; Lummiss et al., 2012). Compounds of this group are economically important and widely used in the flavour and fragrance industries as well as the pharmaceutical and cosmetic industries, and as intermediates in the synthesis of more complex products (Atsumi et al., 2005; Cabral et al., 2014; Aprotosoaie et al., 2016). In recent years, various commercial processes have been developed to obtain these compounds through isomerization of safrole, estragole, and eugenol (Petersen et al., 2010; Rajagopalan et al., 2013; Hassam et al., 2015). Propenylbenzenes have broad biological activities, such as antioxidant, antimicrobial, anti-inflammatory, and antiproliferative actions. For example, essential oils containing isosafrole have shown antioxidant activity and antimicrobial effects on clinical isolates of *Helicobacter pylori*, *Staphylococcus aureus*, and *Escherichia coli* (Bruna et al., 2022). Additionally, safrole oil and its nanoemulgel had an antiproliferative effect on hepatocellular carcinoma cells Hep3B (Eid and Hawash, 2021). Anethole exerts an inhibitory effect on periodontitis by suppressing pro-inflammatory molecules (Moradi et al., 2014; Lal et al., 2022). Anethole-rich oil from *Clausena heptaphylla* leaf has anti-diabetic, tyrosinase-inhibiting, and anti-cholinesterase activities (Lal et al., 2022). Emulsion-encapsulated isoeugenol has antimicrobial effects against food pathogens and spoilage bacteria such as *Listeria monocytogenes*, *Staphylococcus aureus*, *Pseudomonas fluorescens*, and *Leuconostoc mesenteroides* as well as antioxidant and anti-inflammatory properties (Siva et al., 2019).

Given the different biological activities of propenylbenzenes and their widespread use in industry, it is worth looking for derivatives of these compounds. As the interest in compounds obtained *via* environmentally friendly methods is currently growing, the necessity for the development of new methods for producing propenylbenzene derivatives such as diols and hydroxy ketones has increased in recent years. Various methods for obtaining propenylbenzenes and their derivatives have been described in the literature (D’Accolti et al., 1993; Peng et al., 2005; Mecozzi et al., 2017; Nair, 2020; Tentori et al., 2021). In one chemo-enzymatic method, isosafrole is epoxidated and then hydrolysed to yield a stereoisomeric mixture of corresponding diols (Tentori et al., 2021). Peng et al. (2005) presented a method involving selective oxidation of *sec*-1,2-diols, using 2,3-dichloro-5,6-dicyano-1,4-benzoquinone and ultrasound waves to produce α-hydroxy ketones. The oxidation of vicinal diols to α-hydroxy ketones can also be accomplished using hydrogen peroxide (H_2_O_2_) and a manganese catalyst (Mecozzi et al., 2017), dimethyldioxirane or its trifluoromethyl analogue (D’Accolti et al., 1993), or 2-iodoxybenzoic acid (IBX) (Nair, 2020).

Due to the increasing attention paid to green chemistry and the advantages of biotechnological methods, it is important to replace chemical methods with those using biocatalysts in the form of whole cells or isolated enzymes. For instance, Kihumbu et al. (2002) described a method that allowed synthesising all four stereoisomers of 1-phenylpropane-1,2-diol as well as the corresponding hydroxy ketones with high yields starting from benzaldehyde and acetaldehyde using different lyase–alcohol dehydrogenase combinations. Oeggl et al. (2018) reported a method to synthesise hydroxy ketone isomers using lyase or decarboxylase and then 4-methoxyphenyl-1,2-propanediol using alcohol dehydrogenase, with good yields. Finally, the use of benzoylformate decarboxylase or benzaldehyde lyase from *P. putida* and *P. fluorescens* allows the synthesis of hydroxy ketone derivatives of propenylbenzenes (Kurlemann et al., 2009; Kulig et al., 2013; Pérez-Sánchez et al., 2013). However, methods that use whole cells of microorganisms for cost reduction are lacking.

In the presented work, we aimed to obtain oxygenated propenylbenzene derivatives, including diols **1b–5b** and hydroxy ketones **1c–4c**, starting from propenylbenzenes **1a–5a**, using chemo-enzymatic synthesis followed by whole-cell transformation. In addition, biological activity tests were performed on the obtained compounds. This approach allowed us to assess the influence of various chemical groups attached to the propenyl chain and aromatic ring on the investigated biological activities. Based on our previous experience, we decided to test the following biological activities: antimicrobial, antioxidant, haemolytic, and anticancer actions, and the impact on membrane fluidity.

## 2. Materials and methods

### 2.1. Microorganisms

*Bacillus subtilis* PCM2238, *B. subtilis* PCM2850, *Dietzia maris* PCM2292, *Gordonia bronchialis* PCM2167, *Gordonia rubripertincta* PCM2144, *Micrococcus luteus* PCM525, *Pseudomonas aeruginosa* PCM2720, *P. aeruginosa* PCM3035, *Rhodococcus coprophilus* PCM2174, *Rhodococcus erythropolis* PCM2150, *Rhodococcus rhodnii* PCM2157, *Rhodococcus rhodochrous* PCM909, *Rhodococcus ruber* PCM2166, *R. ruber* PCM2171, *R. ruber* PCM2216, *Serratia liquefaciens* PCM2830, *Serratia marcescens* PCM549, *Serratia plumuthica* PCM550, *Serratia* sp. PCM1324, *Streptomyces griseus* subsp. *griseus* PCM2331 were obtained from the Polish Academy of Sciences (Wrocław, Poland)*. Dietzia* sp. DSM44016 and *Rhodococcus erythropolis* DSM44534 were purchased from the German Collection of Microorganisms and Cell Cultures (Braunschweig, Germany). The biocatalysts were maintained at 4°C on PCM medium agar slants. For use in experiments, they were transferred into conical flasks containing PCM medium composed of sodium chlorine (6 g) (Chempur, Piekary Śląskie, Poland), glucose (20 g) (Chempur), casein (2 g) (Biocorp, Warszawa, Poland), bacteriological peptone (10 g) (Biocorp), and yeast extract (2 g) (Chempur) dissolved in distilled water (1 L) at 25°C, pH 5.5.

Fungistatic activity was determined using *Candida albicans* ATCC 90028 from the American Type Culture Collection (ATCC, Manassas, VA, United States), and clinical isolates, *C. albicans* 636/20, *C. albicans* 595/20, and *C. albicans* 38 obtained from Wrocław Medical University, Wrocław, Poland.

### 2.2. Materials

Propenylbenzenes: isosafrole (**1a**), prop-1-en-1-yl benzene (**2a**), anethole (**3a**), 1,2-dimethoxy-4-prop-1-en-1-yl benzene (**4a**), and isoeugenol (**5a**) were purchased from Sigma-Aldrich Chemical Co. (St. Louis, MO, United States). All chemicals and solvents were purchased from Zentek s.r.l. (Milan, Italy) and used without further purification.

### 2.3. Chemo-enzymatic synthesis

#### 2.3.1. Chemo-enzymatic synthesis of diols as substrates for biotransformations

Vicinal diols 1-(1,3-benzodioxol-5-yl)propane-1,2-diol (**1b**), 1-phenylpropane-1,2-diol (**2b**), 1-(4-methoxyphenyl)propane-1,2-diol (**3b**), 1-(3,4-dimethoxyphenyl)propane-1,2-diol (**4b**), and 1-(4-hydroxy-3-methoxyphenyl)propane-1,2-diol (**5b**) were obtained as mixtures of two racemic diastereoisomers by chemo-enzymatic synthesis according to the procedure herein exemplified (Tentori et al., 2021).

Propenylbenzenes **1a–5a** (2.96 mmol) were dissolved in EtOAc (15 mL). A 35% w/w aqueous solution of H_2_O_2_ (382 μL, 4.44 mmol) and Novozym 435 (10 mg) was added to the solution, which was incubated in a thermoshaker at 30°C for 18 h. Then, the enzyme was filtered out and the reaction was quenched first with Na_2_SO_3_, then with a saturated NaHCO_3_ solution. Extraction with EtOAc afforded an organic phase that was dried (Na_2_SO_4_) and concentrated under reduced pressure. The resulting residue was dissolved in MeOH (15 mL), and an excess of KOH (250 mg, 1.5 equiv.) was added to the solution. The reaction was left under magnetic stirring at room temperature for 24 h. The solution volume was reduced to one-third under vacuum, poured into diluted H_2_SO_4_ solution, and extracted with EtOAc. The organic phase was dried (Na_2_SO_4_) and concentrated under vacuum. Trituration of the solid residue with hexane/EtOAc (8,2) afforded mixtures of (R*,S*)- and (R*,R*)-diols **1b–5b**. The structure of the final compounds and the diastereoisomeric ratio, in which they were obtained, were established by GC and NMR analyses.

#### 2.3.2. Tempo-mediated oxidation of diols to corresponding diketones and hydroxy ketones

In order to obtain reference compounds for the identification of products after biotransformation, the following oxidation reaction was implemented. A mixture of corresponding diol **1-4b** (4.9 mmol), TEMPO (8.0 mg, 0.049 mmol) and NaCl (3 mg, 0.049 mmol) in toluene (45 mL) was stirred at 100°C for 4–5 h. The reaction mixture was poured into water and extracted with ethyl acetate. The organic phase was dried and concentrated under reduced pressure to give the corresponding diketone and hydroxy ketone derivatives (approximately 1.8 g from a mixture of each diol). The extract obtained in this way was then purified and allowed to obtain GC/MS chromatograms, ^1^H and ^13^C NMR spectra of corresponding diketones and hydroxy ketones.

### 2.4. Whole-cell biotransformations

#### 2.4.1. Screening-scale biotransformations

Forty millilitres of PCM medium was added into 100-mL tapered flasks and sterilized at 121°C under a pressure of 1 atm. The medium was inoculated with 0.5 mL of pre-cultured bacteria at OD_600_ = 0.3–0.5. The bacterial cultures were incubated at 22°C under shaking at 150 rpm for 3 days. Then, 0.001 g of diols **1b–5b** dissolved in 0.5 mL of dimethyl sulfoxide (DMSO) was added into the flasks. For simple extraction, ethyl acetate (3 mL) was added to the samples (5 mL) in Falcon tubes and shaken at 200 rpm for 5 min. The organic phase was transferred to a vial and dehydrated with anhydrous MgSO_4_. Then, it was filtered through a filter paper into a GC vial. Biotransformation was controlled after 3, 7, and 11 days by GC.

#### 2.4.2. Preparative biotransformations

Five hundred millilitres of PCM medium was added into a 2,000-mL Erlenmeyer flask and sterilized at 121°C for 15 min. The medium was inoculated with 5 mL of pre-prepared bacterial cultures at OD_600_ = 0.3–0.5. The cultures were incubated at 22°C under shaking at 150 rpm for 3 days. Then, 0.2 g of diols **1b–5b** dissolved in 5 mL of DMSO was added into the cultures. The samples were extracted after 3, 7, 11 days and evaluated by GC to estimate the progress of the biotransformation.

### 2.5. Evaluation of biological activity

#### 2.5.1. Fungistatic activity assay

All compounds were tested for biological activity against *C. albicans* ATTC 90028, *C. albicans* 636/20, *C. albicans* 595/20, and *C. albicans* 38 using the broth microdilution method. YPD medium (20 g glucose, 20 g Bacto Peptone, 10 g yeast extract, and 1 L of distilled water, pH 6.5) was used for the tests. The compound solutions were prepared in DMSO and diluted in YPD to obtain final concentrations in the range of 10–250 μg/mL. One hundred microlitres of each solution was pipetted into the wells of a 96-well microtiter plate. The inoculum was standardized to 0.5 McFarland standard and then diluted to obtain a final suspension with a cell density of 0.5–2.5 × 10^3^ CFU/mL. The inoculum size was 100 μL. The positive control comprised DMSO added in the same concentration as the tested compounds in the inoculum, whereas the negative control consisted of DMSO diluted in the broth without the addition of inoculum. The microtiter plates were incubated in a Biosan PST-60 HL Plate Shaker-Thermostat (Riga, Latvia) at 35°C under shaking at 1,000 rpm for 24 h. The fungistatic activity of the compounds was assessed by measuring the absorbance at a wavelength of 595 nm (Epoch; BioTek, Winooski, VT, United States) to determine the MIC_50_ value (i.e., the concentration of a compound required to inhibit the growth of 50% of the microorganisms).

#### 2.5.2. Free-radical scavenging assay

The antiradical activity of all compounds was tested using the 2,2-diphenyl-1-picrylhydrazyl (DPPH) test according to a previously described method (Herald et al., 2012; Dudek et al., 2022). One litre of a fresh solution of 190 μM DPPH in pure methanol was prepared in a dark glass bottle. The solution was kept protected from light and used within a week. The tested compounds and ascorbic acid as a positive control were prepared at concentrations in the range of 5–100 μg/mL. Two hundred microlitres of the test compound dilutions was added to each well of a 96-well microtiter plate. Then, 800 μL of a 190 μM solution of DPPH in methanol was added. DPPH was also added to the negative control, in which 200 μL of methanol was added instead of the test compounds. All samples were tested at least in triplicate. The microtiter plates were incubated in the Biosan PST-60 HL thermoshaker at 25°C under shaking at 1,000 rpm for 1 h. The antiradical activity of the compounds was assessed by measuring the absorbance at 517 nm (Epoch) to determine the EC_50_ value (i.e., the concentration of a compound that gives 50% maximal response). EC_50_ values were calculated using Quest Graph™ EC50 Calculator (AAT Bioquest, Inc.).

#### 2.5.3. Haemolytic activity assay

Haemolytic activity was tested using the method described by Włoch et al. (2020) with minor modifications. This test is based on comparison of the cytotoxicity of all tested compounds in human red blood cells (RBCs) with that in a control group. RBCs for this method were obtained from a blood center from examined and healthy donors. The compounds were tested at 10, 20, 40, 60, 80, and 100 μM concentrations. The control sample contained only ethanol in the same volume as the test samples. The final haematocrit in the test samples was 1.2%. The samples were incubated at 37°C for 1 h. Then, the absorbance at 540 nm was measured using a UV–Vis spectrophotometer (Specord 40, Analytik Jena, Jena, Germany). The haemolytic activity of the compounds was determined as the ratio of the absorbance of haemoglobin in the test samples to that in completely haemolyzed cells multiplied by 100%.

#### 2.5.4. Fluorescence spectroscopy of the RBCs membrane

Using the DPH probe, the anisotropy values of cell membranes of RBCs modified with the test compounds were measured. RBCs for this analysis were prepared according to the method described in subsection 2.5.3. Then, the DPH probe was added to RBCs with a haematocrit of 0.2%. The final concentration of the probe in the sample was 1.3 μM. The mixture was incubated protected from light at 37°C for 30 min. Then, the compounds dissolved in ethanol were added. The compounds were tested at 20, 60, and 100 μM concentrations. Samples with compounds and control samples with alcohol of the appropriate concentration were incubated at 37°C for 1 h. Measurements in triplicate were made in quartz cuvettes using a CARY Eclipse fluorimeter (Varian, San Diego, CA, United States) at 37°C. The excitation wavelength for the DPH probe is λ_exc_ = 360 nm and the emission wavelength is λ_em_ = 426 nm. Based on the changes in DPH on the intensity under polarized light, the anisotropy value was determined according to the formula used in our previous publication (Pruchnik et al., 2018).

#### 2.5.5. Cell culture

The human HepG2 and Caco-2 cell lines were obtained from the American Type Culture Collection (HB-8065™ and HTB-37™) and the MG-63 cell line was obtained from the European Collection of Authenticated Cell Cultures (Merck, Poznań, Poland). HepG2 cells were cultured in Dulbecco’s modified Eagle’s Medium - low glucose (Merck) supplemented with 10% foetal bovine serum (FBS) (Merck). Caco-2 cells were cultured in Dulbecco’s modified Eagle’s medium - high glucose (Merck) supplemented with 10% FBS, 2% HEPES buffer (Thermo Fisher Scientific, Warszawa, Poland), 1% Penicillin–Streptomycin-Amphotericin B Solution (100×, Merck), 1% MEM solution (100×, Thermo Fisher Scientific), and 1% Gentamycin Solution (10 mg/mL, Merck). MG-63 cells were cultured in Minimum Essential Medium (Merck) supplemented with 10% FBS. All cells were cultured in an atmosphere of 5% CO_2_ at 37°C.

#### 2.5.6. Resazurin-based viability assay

HepG2, Caco-2, and MG63 cells were seeded in 96-well plates at 20,000 cells per well, and MG-63 cells at 10,000 cells per well, as described previously (Marycz et al., 2012; Kornicka et al., 2017). The tested compounds were dissolved in ethanol at 10 mg/mL. The concentrations were then adjusted to 1, 50, and 200 μg/mL in culture medium. Four controls were prepared: no ethanol added, and 0.01, 0.5, and 2% EtOH. The cells were treated with the compounds for 24 h. Then, cell viability was evaluated using the resazurin-based assay kit (TOX8), as described previously (Grzesiak et al., 2011; Marycz et al., 2018). The culture medium was replaced with medium containing 10% resazurin dye. The cells were incubated at 37°C in an atmosphere of 5% CO_2_ for 2 h. Absorbance levels were measured spectrophotometrically (Epoch) at a wavelengths of 600 nm for resazurin and 690 nm as a reference.

### 2.6. Chemical analysis procedure

Thin-layer chromatography was conducted using aluminium foil plates coated with silica gel. Compounds were detected by spraying the plates with 1% Ce(SO_4_)_2_ and 2% H_3_[P(Mo_3_O_10_)_4_] in 10% H_2_SO_4_. Gas chromatography (GC; flame ionisation detection, carrier gas H_2_) was carried out on a 7,890 N GC system (Agilent Technologies, Santa Clara, CA, United States) equipped with an HP-5 column (30 m × 0.32 mm × 0.25 μm, Agilent Technologies) according to the following temperature program: 70°C, 300°C (30°C/min) (1 min). Samples (2 μL) were injected with split 9:1; the carrier gas flow was 1 mL/min. The total run time was 9.8 min. Retention times (t_R_) were established as follow: t_R_ = 5.62 min for (1R*,2S*)-**1b** and 5.65 min for (1R*,2R*)-**1b**, t_R_ = 5.5 min for **1c**; t_R_ = 4.16 min for (1R*,2S*)-**2b** and 4.20 min for (1R*,2R*)-**2b**, t_R_ = 3.92 min for **2c**; t_R_ = 5.22 min for (1R*,2S*)-**3b** and 5.26 min for (1R*,2R*)-**3b**, t_R_ = 5.17 min for **3c**; t_R_ = 5.91 min for (1R*,2S*)-**4b** and 5.94 min for (1R*,2R*)-**4b**, t_R_ = 5.89 min for **4c**, and t_R_ = 5.05 min for (1R*,2S*)-**5b** and (1R*,2R*)-**5b**. The structures of the compounds were confirmed using ^1^H nuclear magnetic resonance (NMR) and ^13^C NMR spectra of CDCl_3_ solutions recorded on Avance DRX 600 (600 MHz) and Avance II (400 MHz) spectrometers (Bruker, Billerica, MA, United States). GC–MS analyses were conducted using a HP-5MS column (30 m × 0.25 mm × 0.25 μm) (Agilent Technologies Italia S.p.A., Cernusco sul Naviglio, Italy) with the following temperature program: 60°C (1 min), 150°C (6°C/min) (1 min), 280°C (12°C/min) (5 min).

The NMR spectra of obtained products are as follows:

1-(1,3-benzodioxol-5-yl)propane-1,2-diol as a 3:1 diastereoisomeric mixture of (1R*,2S*) and (1R*,2R*)-**1b**.

(1R*,2S*)-**1b** ^1^H NMR (CDCl_3_, 400 MHz): δ = 6.86–6.84 (m, 1H, Ar–H), 6.81–6.76 (m, 2H, Ar–H), 5.96 (s, 2H, CH_2_), 4.29 (d, 1H, J = 7.4 Hz, CHOH), 3.81 (m, 1H, CHOH), 2.56 (s, 1H, OH), 2.42 (s, 1H; OH), 1.06 (d, 3H, J = 6.3 Hz, CH_3_); ^13^C NMR (CDCl_3_, 100 MHz): δ = 147.91, 147.5, 135.1, 120.5, 108.3, 107.2, 101.1, 79.4, 72.3, 18.9; GC/MS (EI) tr = 21.23 min: m/z (%) = 196 (M^+^, 16), 178 (8), 162 (8), 151 (100), 135 (25), 123 (25).

(1R*,2R*)-**1b** ^1^H NMR (CDCl_3_, 400 MHz): δ = 6.90–6.89 (m, 1H, Ar–H), 6.80–6.78 (m, 2H, Ar–H), 5.95 (s, 2H, CH_2_), 4.57 (d, 1H, J = 4.6 Hz, CHOH), 3.96 (m,1H, CHOH), 2.28 (s, 1H, OH), 1.83 (s, 1H; OH), 1.11 (d, 3H, J = 6.4 Hz, CH_3_); ^13^C NMR (CDCl_3_, 100 MHz): δ = 147.87, 147.3, 134.4, 120.2, 108.2, 107.1, 101.2, 77.5, 71.3, 17.6; GC/MS (EI) tr = 21.31 min: m/z (%) = 196 (M^+^, 16), 178 (8), 162 (8), 151 (100), 135 (25), 123 (25).

1-(1,3-benzodioxol-5-yl)-2-hydroxypropan-1-one **1c** ^1^H NMR (CDCl_3_, 600 MHz): δ = 7.52–7.50 (m, 1H, Ar–H), 7.40–7.42 (m, 1H, Ar–H), 6.86–6.90 (m, 1H, Ar–H), 6.06 (s, 2H, CH_2_), 5.05 (q, 1H, J = 7.0 Hz, CHOH), 1.43 (d, 3H, J = 7.0 Hz, CH_3_); ^13^C NMR (CDCl_3_, 100 MHz): δ = 200.4, 152.6, 148.5, 127.9, 125.2, 108.4, 108.3, 102.2, 69.1, 22.8.; GC/MS (EI) tr = 20.82 min: m/z (%) = 194 (M^+^, 11), 149 (100), 121 (21), 91 (4), 65 (15).

1-(1,3-benzodioxol-5-yl)-1-hydroxypropan-2-one GC/MS (EI) tr = 19.95 min: m/z (%) = 194 (M^+^, 13), 178 (3), 151 (100), 135 (5), 123 (13), 93 (95), 65 (52).

1-(1,3-benzodioxol-5-yl)propane-1,2-dione ^1^H NMR (CDCl_3_, 400 MHz): δ = 7.64–7.62 (m, 1H, Ar–H), 7.49–7.49 (m, 1H, Ar–H), 6.89–6.87 (m, 1H, Ar–H), 6.07 (s, 2H, CH_2_), 2.49 (s, 3H, CH_3_);

^13^C NMR (CDCl_3_, 100 MHz): δ = 200.6, 189.4, 153.0, 148.2, 127.8, 126.2, 108.8, 108.1, 102.0, 26.3.; GC/MS (EI) tr = 19.21 min: m/z (%) = 192 (M^+^, 6), 149 (100), 121 (31), 91 (6), 65 (19).

1-phenylpropane-1,2-diol as a 1.25:1 diastereoisomeric mixture of (1R*,2S*) and (1R*,2R*)-**2b**.

(1R*,2S*)-**2b** ^1^H NMR (CDCl_3_, 400 MHz): δ = 7.39–7.27 (m, 5H, Ar–H), 4.38 (dd, 1H, J^1^ = 7.3 Hz, J^2^ = 2.7 Hz, CHOH), 3.91–3.82 (m, 1H, CHOH), 2.63–2.62 (m, 1H, OH), 2.47–2.46 (m, 1H, OH), 1.07 (d, 3H, J = 6.3 Hz, CH_3_); ^13^C NMR (CDCl_3_, 100 MHz): δ = 128.4, 128.0, 126.6, 125.6, 59.5, 59.0, 17.9.; GC/MS (EI) tr = 14.40 min: m/z (%) = 134 (M^+^ − 18, 3), 134 (3), 117 (7), 108 (100), 103 (2), 91 (17), 79 (93).

(1R*,2R*)-**2b** ^1^H NMR (CDCl_3_, 400 MHz): δ = 7.38–7.33 (m, 5H, Ar–H), 4.69–4.67 (m, 1H, CHOH), 4.06–3.97 (m, 1H, CHOH), 2.38–2.37 (m, 1H,OH), 1.88–1.87 (m, 1H, OH), 1.09 (d, 3H, J = 6.4 Hz); ^13^C NMR (CDCl_3_, 100 MHz): δ = 128.4, 128.0, 126.6, 125.6, 59.5, 59.0, 17.9.; GC/MS (EI) tr = 14.60 min: m/z (%) = 134 (M^+^ − 18, 3), 134 (3), 117 (13), 108 (100), 103 (3), 91 (18), 79 (93).

2-hydroxy-1-phenylpropan-1-one **2c** ^1^H NMR (CDCl_3_, 600 MHz): δ = 7.93–7.91 (m, 2H, Ar–H), 7.93–7.91 (m, 1H, Ar–H), 7.50–7.47 (m, 2H, Ar–H), 5.19–5.14 (q, 1H, J = 7.0 Hz, CHOH), 1.24 (s, 1H, OH), 1.21 (d, 3H, J = 9.5 Hz, CH_3_); ^13^C NMR (CDCl_3_, 100 MHz): δ = 202.5, 134.1, 133.7, 130.3, 128.9, 128.8, 128.6, 69.4, 13.3. GC/MS (EI) tr = 13.07 min: m/z (%) = 135 (M^+^ − 15, 1)*, 135 (1), 105 (100), 77 (43), 51 (12)*.

*1-hydroxy-1-phenylpropan-2-one GC/MS (EI) tr = 12.34 min: m/z (%) = 150 (M^+^, 2), 107 (100), 89 (2), 79 (85)*.

*1-phenylpropane-1,2-dione ^1^H NMR (CDCl_3_, 400 MHz):* δ = 8.02–8.00 (m, 2H, Ar–H), 7.66–7.60 (m, 1H, Ar–H), 7.52–7.48 (m, 2H, Ar–H)*, 2.53 (s, 3H, CH_3_); ^13^C NMR (CDCl_3_, 100 MHz):* δ = 200.5, 191.4, 134.6, 131.8, 130.3, 128.9, 26.4.; GC/MS (EI) tr = 11.23 min: m/z (%) = 148 (M^+^, 4), 105 (*100), 77 (71), 51 (20)*.

1-(4-methoxyphenyl)propane-1,2-diol as a 3:1 diastereoisomeric mixture of (1R*,2S*) and (1R*,2R*)-**3b**.

(1R*,2S*)-**3b** ^1^H NMR (CDCl_3_, 400 MHz) δ = 7.30–7.25 (m, 2H, Ar-H), 6.91–6.87 (m, 2H, Ar-H), 4.33 (dd, 1H, J^1^ = 7.7 Hz, J^2^ = 2.9 Hz, CHOH), 3.89–3.82 (m, 1H, CHOH), 3.81 (s, 3H, OCH_3_), 2.47–2.42 (m, 2H, OH), 1.05 (d, 3H, J = 7.4 Hz, CH_3_); ^13^C NMR (CDCl_3_, 100 MHz): δ = 159.5, 146.6, 133.2, 128.0, 114.0, 79.2, 72.3, 18.8.; GC/MS (EI) tr = 19.75 min: m/z (%): 182 (M^+^, 4), 164 (6), 137 (100), 121 (35), 109 (20), 94 (17).

(1R*,2R*)-**3b** ^1^H NMR (CDCl_3_, 400 MHz) δ = 7.30–7.25 (m, 2H, Ar-H), 6.91–6.87 (m, 2H, Ar-H), 4.61–4.59 (m, 1H, CHOH), 4.01–3.97 (m, 1H, CHOH), 3.81 (s, 3H, OCH_3_), 2.21–2.20 (m, 1H, OH), 1.79–1.78 (m, 1H, OH), 1.11 (d, 3H, J = 6.4 Hz, CH_3_); ^13^C NMR (CDCl_3_, 100 MHz): δ = 159.5, 146.6, 133.2, 128.0, 114.0, 79.2, 72.3, 18.8.; GC/MS (EI) tr = 19.83: m/z (%): 182 (M^+^, 3), 164 (6), 137 (100), 121 (37), 109 (19), 94 (17).

2-hydroxy-1-(4-methoxyphenyl)propan-1-one **3c** ^1^H NMR (CDCl_3_, 600 MHz): δ = 7.51–7.49 (m, 2H, Ar–H), 6.92–6.88 (m, 2H, Ar–H), 5.16–5.06 (m, 1H, CHOH), 3.93 (s, 3H, OCH_3_), 1.43 (d, 3H, J = 7.0 Hz, CH_3_); ^13^C NMR (CDCl_3_, 100 MHz): δ = 201.2, 164.1, 132.9, 132.4, 131.1, 114.2, 113.8, 69.0, 55.6, 22.8; GC/MS (EI) tr = 19.96: m/z (%): 180 (M^+^, 3), 135 (100), 107 (9), 92 (10), 77 (14).

1-hydroxy-1-(4-methoxyphenyl)propan-2-one GC/MS (EI) tr = 18.79: m/z (%): 180 (M^+^, 3), 137 (100), 109 (25), 94 (25), 77 (23).

1-(4-methoxyphenyl)propane-1,2-dione ^1^H NMR (CDCl_3_, 400 MHz): δ = 8.03–8.00 (m, 2H, Ar–H), 6.98–6.94 (m, 2H, Ar-H), 3.89 (s, 3H, OCH_3_), 2.50 (s, 3H, CH_3_); ^13^C NMR (CDCl_3_, 100 MHz): δ = 201.1, 190.0, 164.8, 132.8, 124.7, 114.2, 55.6, 26.5.; GC/MS (EI) tr = 17.63 min: m/z (%) = 178 (M^+^, 2), 135 (100), 107 (11), 92 (16), 77 (22).

1-(3,4-dimethoxyphenyl)propane-1,2-diol as a 3:1 diastereoisomeric mixture of (1R*,2S*) and (1R*,2R*)-**4b**.

(1R*,2S*)-**4b** ^1^H NMR (CDCl_3_, 400 MHz) δ = 6.94–6.84 (m, 3H, Ar-H), 4.32–4.30 (d, 1H, CHOH), 3.89 (s, 3H, OCH_3_), 3.87 (s, 3H, OCH_3_), 3.85–3.81 (m, 1H, CHOH), 2.69 (s, 1H, OH), 2.54 (s, 1H, OH), 1.06 (d, 3H, J = 6.3 Hz, CH_3_); ^13^C NMR (CDCl_3_, 100 MHz): δ = 149.1, 148.9, 133.7, 119.3, 119.1, 111.1, 109.8, 79.3, 72.2, 55.9, 18.8.; GC/MS (EI) tr = 22.22 min: m/z (%) = 212 (M^+^, 2), 194 (24), 178 (9), 167 (12), 151 (100), 139 (8).

(1R*,2R*)-**4b** ^1^H NMR (CDCl_3_, 400 MHz) δ = 6.84–6.82 (m, 3H, Ar-H), 4.59–4.58 (m, 1H, CHOH), 3.89 (s, 3H, OCH_3_), 4.01–3.95 (m, 1H, CHOH), 3.87 (s, 3H, OCH_3_), 2.41 (s, 1H, OH), 1.91 (s, 1H, OH), 1.12 (d, 3H, J = 6.1 Hz, CH_3_); ^13^C NMR (CDCl_3_, 100 MHz): δ = 149.0, 148.9, 133.1, 119.1, 111.0, 109.8, 77.5, 71.4, 55.9, 17.5; GC/MS (EI) tr = 22.22 min: m/z (%) = 212 (M^+^, 2), 194 (24), 178 (9), 167 (12), 151 (100), 139 (8).

1-(3,4-dimethoxyphenyl)-2-hydroxypropan-1-one **4c** ^1^H NMR (CDCl_3_, 600 MHz): δ = 7.52–7.49 (m, 2H, Ar–H), 6.91–6.89 (m, 1H, Ar–H), 5.14–5.08 (m, 1H, CHOH), 3.94 (s, 3H, OCH_3_), 3.93 (s, 3H, OCH_3_), 1.43 (d, 3H, J = 7.1 Hz, CH_3_); ^13^C NMR (CDCl_3_, 100 MHz): δ = 200.9, 154.1, 149.4, 126.2, 123.5, 110.8, 110.2, 68.9, 56.2, 56.1, 23.0.; GC/MS (EI) tr = 22.20 min: m/z (%) = 210 (M^+^, 9), 192 (2), 165 (100), 151 (5), 137 (9), 122 (6).

1-(3,4-dimethoxyphenyl)-1-hydroxypropan-2-one GC/MS (EI) tr = 21.20 min: m/z (%) = 210 (M^+^, 6), 192 (7), 167 (100), 151 (22), 139 (61), 124 (23).

1-(3,4-dimethoxyphenyl)propane-1,2-dione ^1^H NMR (CDCl_3_, 400 MHz): δ = 7.67–7.65 (m, 1H, Ar–H), δ = 7.58–7.76 (m, 1H, Ar–H), 6.92–6.90 (m, 1H, Ar–H), 3.97 (s, 3H, OCH_3_), 3.94 (s, 3H, OCH_3_), 2.51 (s, 3H, CH_3_); ^13^C NMR (CDCl_3_, 100 MHz): δ = 200.9, 190.0, 154.7, 149.3, 126.6, 124.6, 111.0, 110.2, 56.0, 55.9, 26.5.; GC/MS (EI) tr = 21.00 min: m/z (%) = 208 (M^+^, 3), 165 (100), 137 (9), 122 (8).

1-(4-hydroxy-3-methoxyphenyl)propane-1,2-diol as a 3:1 diastereoisomeric mixture of (1R*,2S*) and (1R*,2R*)-**5b**.

(1R*,2S*)-**5b** ^1^H NMR (CDCl_3_, 400 MHz) δ = 6.94–6.87 (m, 3H, Ar-H), 5.62 (s, 1H, Ar-OH), 4.31 (d, 1H, J = 7.5 Hz, CHOH), 3.90 (s, 3H, OCH_3_), 3.87–3.81 (m, 1H, CHOH), 1.06 (d, 3H, J = 6.3 Hz, CH_3_); ^13^C NMR (CDCl_3_, 100 MHz): δ = 146.7, 145.6, 133.0, 120.0, 114.3, 109.0, 79.4, 72.3, 56.0, 18.9.; GC/MS (EI) tr = 21.71 min: m/z (%) = 198 (M^+^, 12), 180 (9), 153 (100), 137 (40), 125 (19).

(1R*,2R*)-**5b** ^1^H NMR (CDCl_3_, 400 MHz) δ = 6.84–6.80 (m, 3H, Ar-H), 5.62 (s, 1H, Ar-OH), 4.57 (d, 1H, *J* = 4.8 Hz, CHOH), 3.99–3.96 (m, 1H, CHOH), 3.90 (s, 3H, OCH_3_), 1.12 (d, 3H, *J* = 6.4 Hz, CH_3_); ^13^C NMR (CDCl_3_, 100 MHz): δ = 146.0, 145.4, 132.4, 119.9, 114.2, 109.2, 77.6, 71.4, 56.0, 17.6.; GC/MS (EI) tr = 21.71 min: m/z (%) = 198 (M^+^,12), 180 (9), 153 (100), 137 (40), 125 (19).

### 2.7. Statistical analysis

The biotransformation and biological activity experiments were performed in triplicate, and the data are presented in tables and figures as means and standard deviations. Student’s t-test was used to compare the means. Differences with *p* < 0.05 were considered significant. Statistical analyses were performed using Past 4.02 (Oyvind Hammer).

## 3. Results and discussion

**Figure 1:**
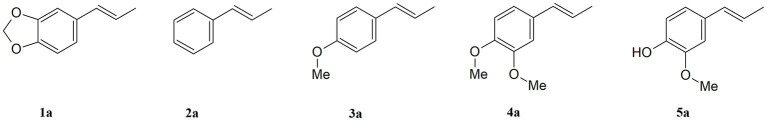
Structures of the studied propenylbenzenes **1a–5a**.

In this research, we focused on propenylbenzenes **1a–5a** (Figure 1), as these compounds have been reported to possess various biological activities, such as antimicrobial, antioxidant, and antiproliferative activities (Moradi et al., 2014; Siva et al., 2019; Bruna et al., 2022; Lal et al., 2022). Taking this into consideration, several derivatives of these compounds were obtained in order to study the influence of their structure on biological activity.

**Figure 2:**
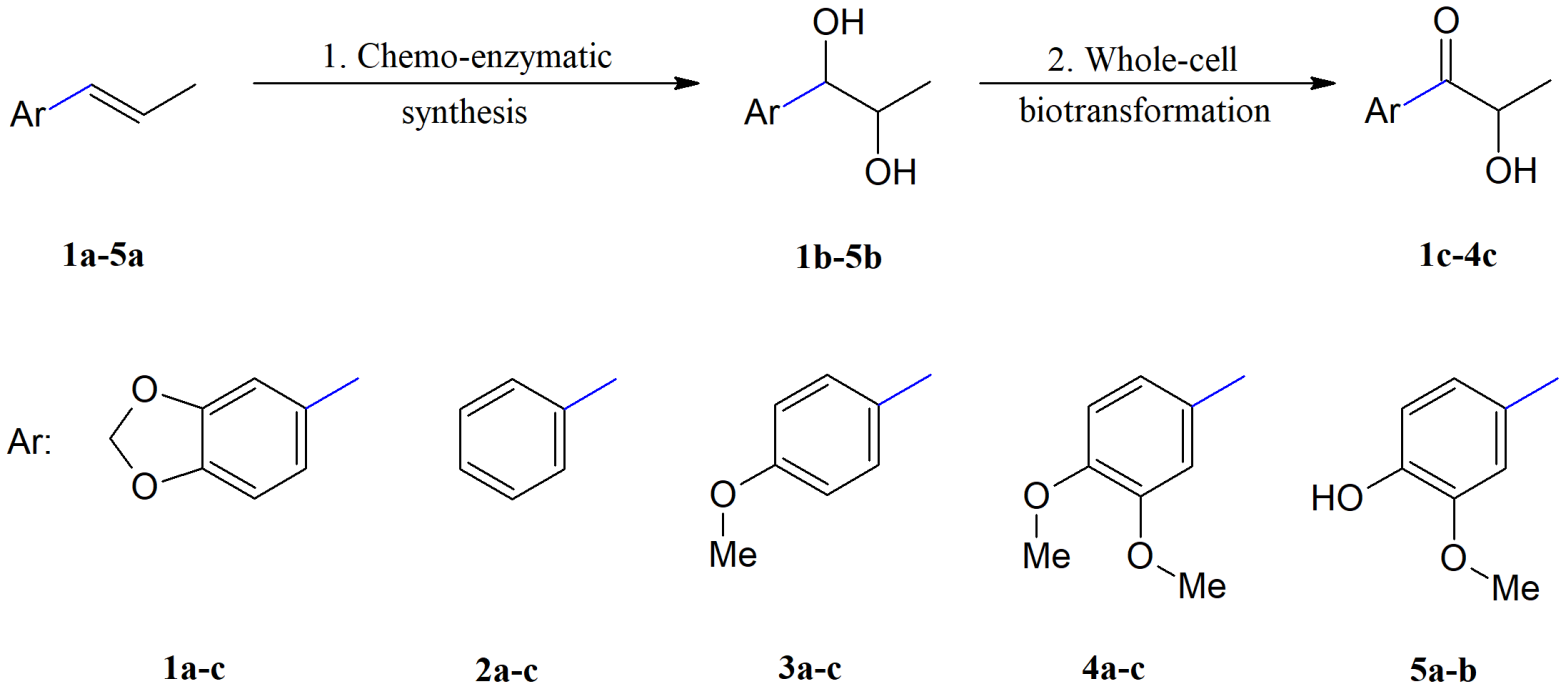
Two-step synthesis of propenylbenzene derivatives: diols **1b–5b** and hydroxy ketones **1c–4c**. 1. aq. H_2_O_2_, Novozym 435, EtOAc, 30°C, 18 h, KOH, MeOH; 2. PCM medium, 23°C, 3–11 days.

The two-step biocatalytic synthesis (Figure 2) of oxygenated propenylbenzene derivatives involved (1) chemo-enzymatic epoxidation followed by hydrolysis of the starting compounds **1a–5a** to the corresponding diols **1b–5b** and (2) microbial oxidation of the diols **1b–5b** into hydroxy ketones **1c–4c**. The thus-obtained propenylbenzenes derivatives were tested for their antimicrobial, antioxidant, haemolytic, and anticancer activities and their impact on membrane fluidity.

### 3.1. Chemo-enzymatic synthesis of diols

In the first step, compounds **1a–5a** were converted into the corresponding diols accordingly to the method recently described by Tentori et al. (2021) for isosafrole (**1a**). This method is based on the Prilezhaev reaction of isosafrole with a peroxycarboxylic acid obtained *in situ* by lipase-catalysed perhydrolysis of the corresponding carboxylic acid in the presence of H_2_O_2_. We used aqueous H_2_O_2_ and commercial immobilized lipase B from *Candida antarctica* (Novozym 435) based on a report by Björkling et al. (1990). We modified this method, using ethyl acetate, which undergoes lipase-mediated perhydrolysis, resulting in the formation of ethanol and peroxyacetic acid. This avoided the addition of octanoic acid and other carboxylic acids to promote the reaction. However, after applying this method to compound **1a**, we noticed that diol **1b** was formed only in small amounts, whereas the dominant products were epoxide and monoacetate derivatives. Therefore, methanolic KOH was added to the compound mixture, which allowed for complete oxirane ring cleavage and promoted monoacetate hydrolysis. As a result of this reaction, epoxide and the monoacetate derivatives were readily converted into vicinal diol **1b**, with a high yield (69%). Considering these results, we decided to apply this method also to the other structurally similar propenylbenzenes **2a–5a**, which allowed to obtain corresponding diols **2b–5b** for use as substrates in biotransformation processes. As a result of implementing the aforementioned method, a diasteroisomeric mixture of (R*,S*)- and (R*,R*)-diols **1b–5b** was obtained, with high isolation yields in the range of 65–80%. Compared to other methods described in the literature, the method we proposeis highly efficient, using an immobilized biocatalyst that can be easily recovered from the reaction by filtration, and employing cheap H_2_O_2_ as an oxidant. Other enzymatic methods described so far (Kihumbu et al., 2002; Oeggl et al., 2018) were aimed at obtaining pure enantiomers of selected propenylbenzenes, whereas in our method we focused on obtaining diols as racemic diastereoisomers to be submitted to further microbial transformations.

### 3.2. Biotransformations of diols

Screening-scale whole-cell transformations of diols **1b–5b** were conducted using 22 bacterial strains belonging to the genera *Bacillus*, *Dietzia*, *Gordonia*, *Micrococcus*, *Pseudomonas*, *Rhodococcus*, *Serratia*, and *Streptomyces*. In biotransformations with diols **1b–4b**, corresponding hydroxy ketones **1c–4c** were formed as the main products. In addition, small amounts of diketones and isomeric forms of hydroxy ketones were detected in the reaction mixture (data are shown in the Supplementary Tables S1–S4). The most efficient biocatalysts (*Dietzia* sp. DSM44016, *R. erythropolis* PCM2150, *R. erythropolis* DSM44534, and *R. ruber* PCM2166) in the screening-scale experiments are listed in Table 1. In all biotransformations, the amount of hydroxy ketones **1c–4c** (determined by GC) clearly increased over time up to day 11. The highest percentage of product **1c** (85%) was observed in the biotransformation with *Dietzia* sp. DSM44016. In contrast, hydroxy ketones **2c** and **3c** were the most efficiently produced in the biotransformation with *R. ruber* PCM2166, and a high amount of product **4c** (77–79%) was obtained in the biotransformations conducted by *R. erythropolis* DSM44534 and *R. ruber* PCM2166. All strains mentioned effectively produced hydroxy ketones, except for the biotransformation of compound **3b** with strain *R. erythropolis* PCM2150, after which only 38% hydroxy ketone **3c** was detected. Due to the small differences in biotransformations with these bacteria, all strains were selected for preparative scale biotransformations, which allowed to isolate products and determine their structures by NMR analysis. Biotransformation of diol **5b** did not yield hydroxy ketones or other products; therefore, this substrate was not studied on a preparative scale.

**Table 1:** Selected results of the screening-scale biotransformations of diols **1b–4b**.

Strain	Time (days)	**1c** (%)	**2c** (%)	**3c** (%)	**4c** (%)
*Dietzia* sp. DSM44016	3	48 (±2.1)	45 (±4.1)	12 (±1.5)	39 (±3.6)
	7	66 (±3.3)	70 (±2.9)	41 (±1.9)	58 (±4.7)
	11	85 (±3.9)	74 (±3.4)	74 (±4.3)	69 (±1.9)
*Rhodococcus erythropolis* PCM2150	3	30 (±3.5)	19 (±1.0)	18 (±2.6)	25 (±3.3)
	7	46 (±4.2)	46 (±2.4)	27 (±3.1)	51 (±4.2)
	11	63 (±2.9)	67 (±4.1)	38 (±3.0)	71 (±2.1)
*Rhodococcus erythropolis* DSM44534	3	53 (±3.3)	24 (±1.9)	27 (±3.2)	27 (±1.7)
	7	72 (±1.3)	52 (±3.4)	61 (±3.0)	52 (±2.4)
	11	73 (±3.6)	70 (±2.1)	82 (±2.0)	79 (±2.9)
*Rhodococcus ruber* PCM2166	3	40 (±3.1)	24 (±2.1)	38 (±4.2)	34 (±1.8)
	7	45 (±4.2)	48 (±2.8)	64 (±3.1)	51 (±2.5)
	11	62 (±2.6)	87 (±3.3)	88 (±2.1)	77 (±2.0)

%, determined by GC.

When planning the scale-up process, we decided to add substrates **1b–4b** in an amount of 0.2 g each and to conduct the biotransformation process for 11 days (Table 2). During preparative-scale biotransformation of substrate **1b**, the highest amount of product **1c** (0.125 g, yield = 62.5%) was obtained using *Dietzia* sp. DSM44016. In the course of biotransformation of diol **2b**, only *R. ruber* PCM2166 catalysed the reaction, affording 0.081 g of **2c** (yield = 40.5%). The high concentration of substrate **2b** had an inhibitory effect on all other strains, and only 10–24% of hydroxy ketone **2c** and unreacted substrate were detected in the samples. The highest amount of hydroxy ketone **3c** (0.115 g, yield = 57.5%) was obtained with *R. erythropolis* DSM44534. Finally, *R. erythropolis* PCM2150 yielded 0.119 g (59.5%) of product **4c**.

**Table 2:** Summary of the biotransformations of propenylbenzenes **1a–4a** performed on a preparative scale after 11 days.

Strain	Substrate	Product	Isolation yield (%)	Amount of product (g)
*Dietzia* sp. DSM44016	**1b**	**1c**	62.5	0.125
*R. erythropolis* PCM2150			45.5	0.091
*R. erythropolis* DSM44534			36	0.072
*R. ruber* PCM2166	**2b**	**2c**	40.5	0.081
*Dietzia* sp. DSM44016	**3b**	**3c**	44.5	0.089
*R. erythropolis* PCM2150			36.5	0.073
*R. erythropolis* DSM44534			57.5	0.115
*Dietzia* sp. DSM44016	**4b**	**4c**	48.5	0.097
*R. erythropolis* PCM2150			59.5	0.119
*R. erythropolis* DSM44534			42.5	0.085

During the preparative-scale biotransformations, we noticed that an increase in the substrate concentration significantly affected the course of the process with selected bacterial strains. In the case of *Dietzia* sp. DSM44016, the highest yields were achieved in the biotransformation of substrate **1b**, which has a dioxolane group in its structure, whereas hydroxy ketones **3c** and **4c** (with one and two methoxy groups, respectively) were obtained with lower yields. However, diol **2b** (without additional substituent) was toxic to this strain and only small amount of product **2c** was detected by GC. These findings indicated that the dioxolane, methoxy, and dimethoxy groups reduce the toxicity of diols derived from propenylbenzenes on *Dietzia* sp. DSM44016. Biotransformation with *R. ruber* PCM2166 yielded a significantly smaller amount of compound **2c**, and with substrates **1b** and **3b–4b**, no products were formed. This indicates that *R. ruber* PCM2166 accepts only propenylbenzene diol derivatives without aromatic ring substituents. Biotransformations with *R. erythropolis* DSM44534 and *R. erythropolis* PCM2150 were characterized by a similar course of bio-oxidation, which is not surprising considering that they belong to the same species. These microorganisms effectively catalysed the oxidation of diols **1b** and **3b–4b** to corresponding hydroxy ketones **1c** and **3c–4c**. The high concentration of substrate **2b** had an inhibitory effect on these strains, as observed with *Dietzia* sp. DSM44016.

Several methods for the synthesis of hydroxy ketones **1c–4c** have been reported (Kihumbu et al., 2002; Peng et al., 2005; Kurlemann et al., 2009). In one method using 2,3-dichloro-5,6-dicyano-1,4-benzoquinone and ultrasound waves, compound **1c** was obtained with a 72% yield (Peng et al., 2005). Enzymatic reaction with benzaldehyde lyase or benzoylformate decarboxylase afforded hydroxy ketone **2c** with a yield of 95% (Kihumbu et al., 2002). Hydroxy ketone **3c** (yield = 81–84%) was obtained in a two-step process involving oxidation of *trans*-anethol using *Trametes hirsuta* lyophilisate followed by ligation of *para*-anisaldehyde with acetaldehyde using benzaldehyde lyase or benzoylformate decarboxylase (Kurlemann et al., 2009). The biocatalytic method presented in this paper is an attractive alternative to the methods described in the literature. It does not require expensive reagents and enzymes and allows to work starting from propenylbenzenes, most of which can be recovered from renewable feedstocks (natural essential oils and plants extracts).

### 3.3. Fungistatic activity

The fungistatic activities of propenylbenzenes **1a–5a** have been described in the literature (Moradi et al., 2014; Siva et al., 2019; Bruna et al., 2022; Lal et al., 2022). The MIC values of these compounds range from 100 to 400 μg/mL, with the lowest value reported for isosafrole (**1a**) and the highest for isoeugenol (**5a**) and its derivative **4a** (Kubo et al., 1993). These studies were conducted using *C. albicans* ATTC18804, and it should be noted that MIC_50_ values were not calculated. Additionally, studies using the disc diffusion method for *C. albicans* ATCC 66027 and the microdilution method (with a different cell density) for *C. albicans* ATCC 22019 showed that anethole (**3a**) and essential oil containing isosafrole (**1a**) have antifungal activity (Moradi et al., 2014; Bruna et al., 2022; Lal et al., 2022). The antimicrobial activities of isosafrole (**1a**), anethol (**3a**), and isoeugenol (**5a**) have been reported; however, antimicrobial activities of their derivatives, such as diols and hydroxy ketones, have not been reported. Therefore, we assessed how the structures of these compounds affect their biological activity (Moradi et al., 2014; Siva et al., 2019; Bruna et al., 2022; Lal et al., 2022). In our previous studies on fungistatic activity, we used yeasts of the genus *Candida* (Gach et al., 2021; Krężel et al., 2022) therefore, we used *C. albicans* strains as the test model in the current study.

**Figure 3:**
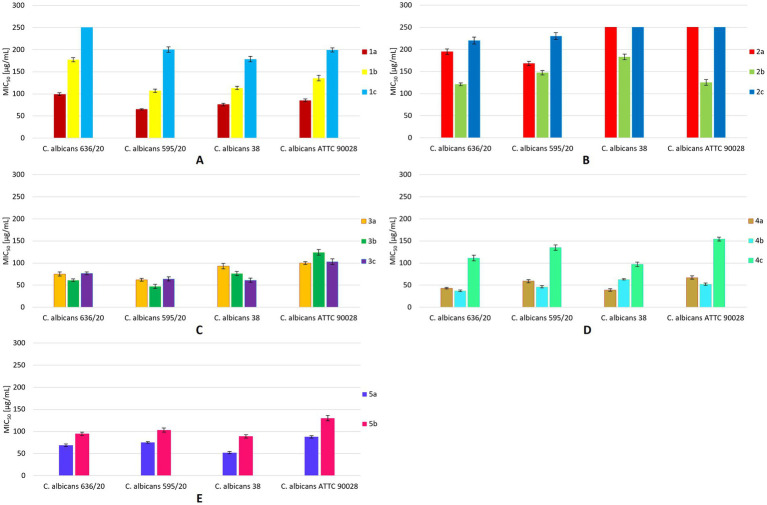
MIC_50_ values [μg/mL] for all tested compounds against *C. albicans* strains. The results are shown as mean values ± standard deviations.

Isosafrole (**1a**) efficiently inhibited the growth of *C. albicans* strains 636/20, 595/20, 38, and ATTC90028, with MIC_50_ values below 100 μg/mL (Figure 3A). The highest inhibitory activity (MIC_50_ = 65 μg/mL) of this compound was noted for *C. albicans* 595/20. The fungistatic activity of dihydroxy derivative **1b** was decreased compared to that of the starting compound **1a**. The MIC_50_ values of diol **1b** for *C. albicans* 595/20 and 636/20 were 107 and 177 μg/mL, respectively. Compared to those of **1a** and **1b**, the inhibitory activity of hydroxy ketone **1c** against *C. albicans* strains 595/20, 38, and ATTC90028 was substantially decreased (MIC_50_ = 178–200 μg/mL). Compound **1c** showed no inhibitory activity against *C. albicans* 636/20, even at a concentration of 250 μg/mL. The presence of hydroxy and carbonyl groups in compounds **1b–c** suppressed their fungistatic activity against all tested strains, whereas the presence of two hydroxy groups in compounds **2a–c** increased their fungistatic activity against all tested strains (Figure 3B). Compared to **2a** and **2c**, diol **2b** showed the highest inhibitory activity against all tested strains, with the lowest noted for *C. albicans* 636/20 (MIC_50_ = 121 μg/mL) and ATTC90028 (MIC_50_ = 125 μg/mL). The growth of *C. albicans* 38 and ATTC90028 was not affected by compounds **2a** and **2c**, even at concentrations above 250 μg/mL. Anethole (**3a**) exhibited significant inhibitory activity against all tested strains, with MIC_50_ values of 62–100 μg/mL and the highest activity noted for *C. albicans* 595/20 (MIC_50_ = 62 μg/mL) (Figure 3C). Diol **3b** showed increased fungistatic activity against *C. albicans* 595/20, 636/20, and 38 (MIC_50_ = 47–76 μg/mL), with the lowest MIC_50_ of 47 μg/mL for *C. albicans* 595/20. Hydroxy ketone **3c** showed increased fungistatic activity against *C. albicans* 38 (MIC_50_ = 61 μg/mL). Compounds **3a–c** showed variable fungistatic activity against the tested strains; therefore, the impacts of their structures on fungistatic activity were difficult to determine. Among compounds **4a–c**, **4a** (MIC_50_ = 39–67 μg/mL) and **4b** (MIC_50_ = 37–63 μg/mL) showed the highest fungistatic activity against all strains, with diol **4b** showing higher activity against *C. albicans* 636/20, 595/20, and ATTC90028 (Figure 3D). The presence of a carboxyl group in compound **4c** substantial suppressed its fungistatic activity. Isoeugenol (**5a**) showed substantially higher fungistatic activity than its diol derivative **5b** against all four strains (MIC_50_ = 52–88 μg/mL) (Figure 3E). By comparing the various compounds and their derivatives, we noticed that the presence of additional substituents in the aromatic ring of the starting propenylbenzenes **1a**, **3a**, **4a**, and **5a** had a positive effect on their fungistatic activity when compared to that of **2a**. The presence of hydroxy groups in diols **2b–4b** had a positive effect on their inhibitory activity against most strains tested when compared with that of the starting compounds **2a–4a**. All hydroxy diols, except **3c**, tended to be less active than the starting compounds and diols.

### 3.4. Antiradical activity

The antiradical activities of some propenylbenzenes or essential oils containing them have been reported in the literature. Eid and Hawash (2021) reported that safrole (the isomer of compound **1a**) exhibits antioxidant activity, with an IC_50_ value of 50.28 μg/mL. A study on the antiradical activity of isosafrole (**1a**) in essential oils containing a certain amount of this compound (19.5%) revealed that such mixtures of different compounds exhibit IC_50_ values >1,000 μg/mL (Bruna et al., 2022). For anethol (**3a**), an EC_50_ value of 8.69 has been reported (Lal et al., 2022). According to Siva et al. (2019), the antioxidant activity of isoeugenol (**5a**) was 86%.

**Figure 4:**
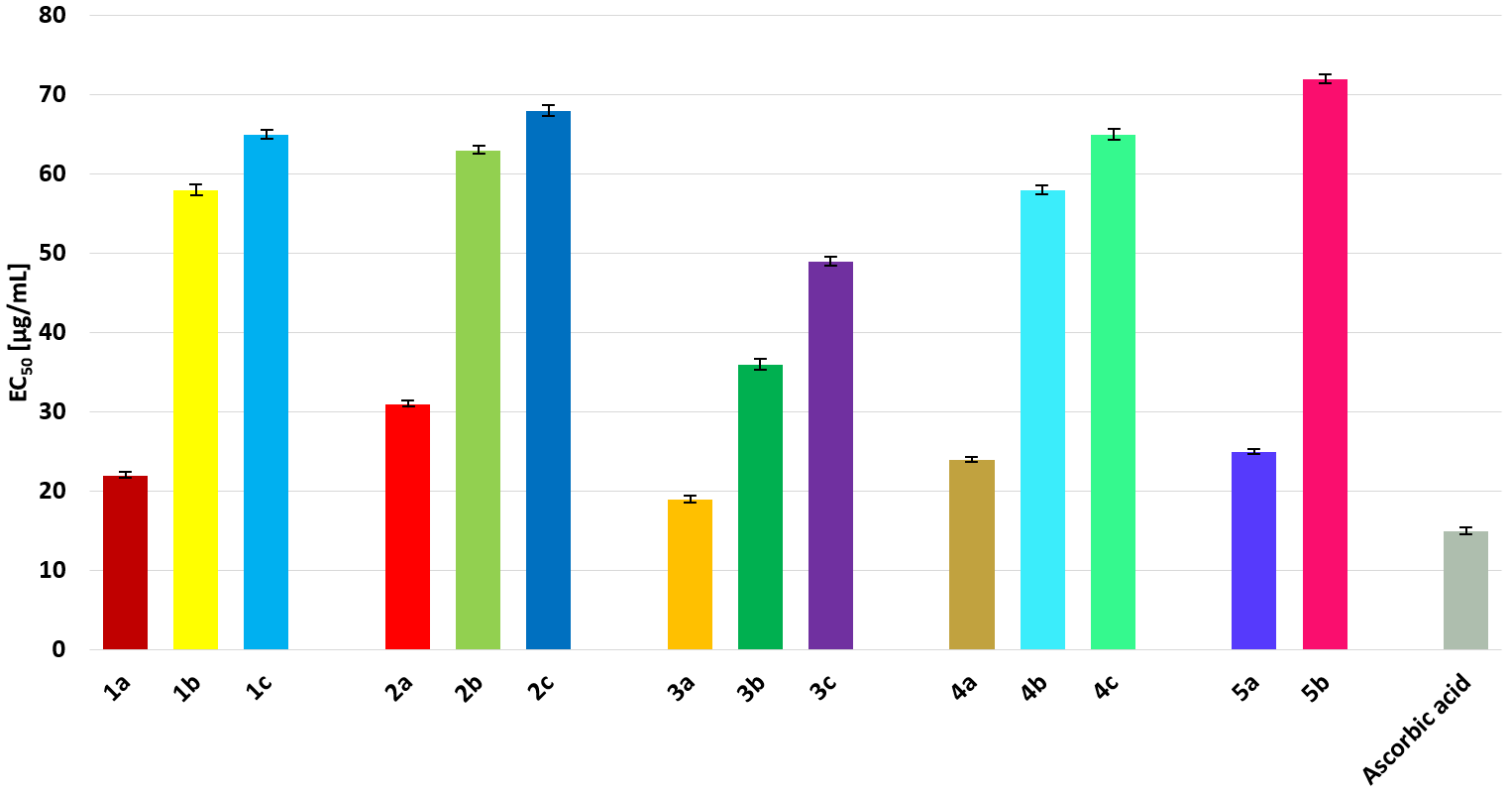
EC_50_ values [μg/mL] for all tested compounds in comparison to ascorbic acid. The results are shown as mean values ± standard deviations.

The antioxidant activities of all compounds were compared with that of the standard, ascorbic acid, to estimate their free radical-scavenging power. The lowest EC_50_ value was noted for ascorbic acid (15.21 μg/mL), which was used as a positive control (Figure 4). Among the tested compounds, those with a double bond in the propenyl group had the lowest EC_50_ values. Among these, anethole (**3a**) showed the highest antiradical activity (EC_50_ = 19.13 μg/mL). Significantly lower antioxidant activity was observed for diols **1b–5b** and hydroxy ketones **1c–4c**, with EC_50_ values ranging from 36.34 to 72.08 μg/mL. Among the diols, **3b** showed the highest antiradical activity. All hydroxy ketones showed lower activity than the starting compounds and diols.

### 3.5. Haemolytic activity

The degree of cytotoxicity of the compounds was evaluated using a haemolytic activity assay, in which the haemolytic activity of 13 compounds at 10–100 μM on human RBCs was evaluated. According to toxicity classification, compounds are highly toxic if the haemolysis rate is 90–100% and nontoxic if the haemolysis rate is 0–9% (Pagano and Faggio, 2015). Data shown in the Supplementary Table S5 show the percentage of haemolysis after a 1 h incubation with the compounds at various concentrations at 37°C. The percentage of haemolysis was similar to that of the control for all compounds and did not exceed 3%; therefore, oxygenated derivatives of propenylbenzenes do not cause haemolysis of RBCs. Our results indicate that the compounds in the range of used concentrations do not have a toxic effect on human red blood cells.

The results obtained for compounds **1a** and **5a** are in agreement with the results of other authors, in which it was shown that derivatives of safrole or eugenol present negligible hemolytic capacity (Hidalgo and De la Rosa, 2009; Madrid et al., 2014). However, all derivatives of safrole exhibited haemolytic activity (minor than 10%), and for the derivatives of eugenol activity lower than 1% was detected, which indicate their non-toxicity. Other authors have also shown that isoeugenol has low cytotoxic activity (Bhatia et al., 2011). Due to the fact that compounds **1-5b** and **1-4c** were described for the first time, the results obtained for these compounds are novel.

### 3.6. Effects of the compounds on RBCs membrane fluidity

**Figure 5:**
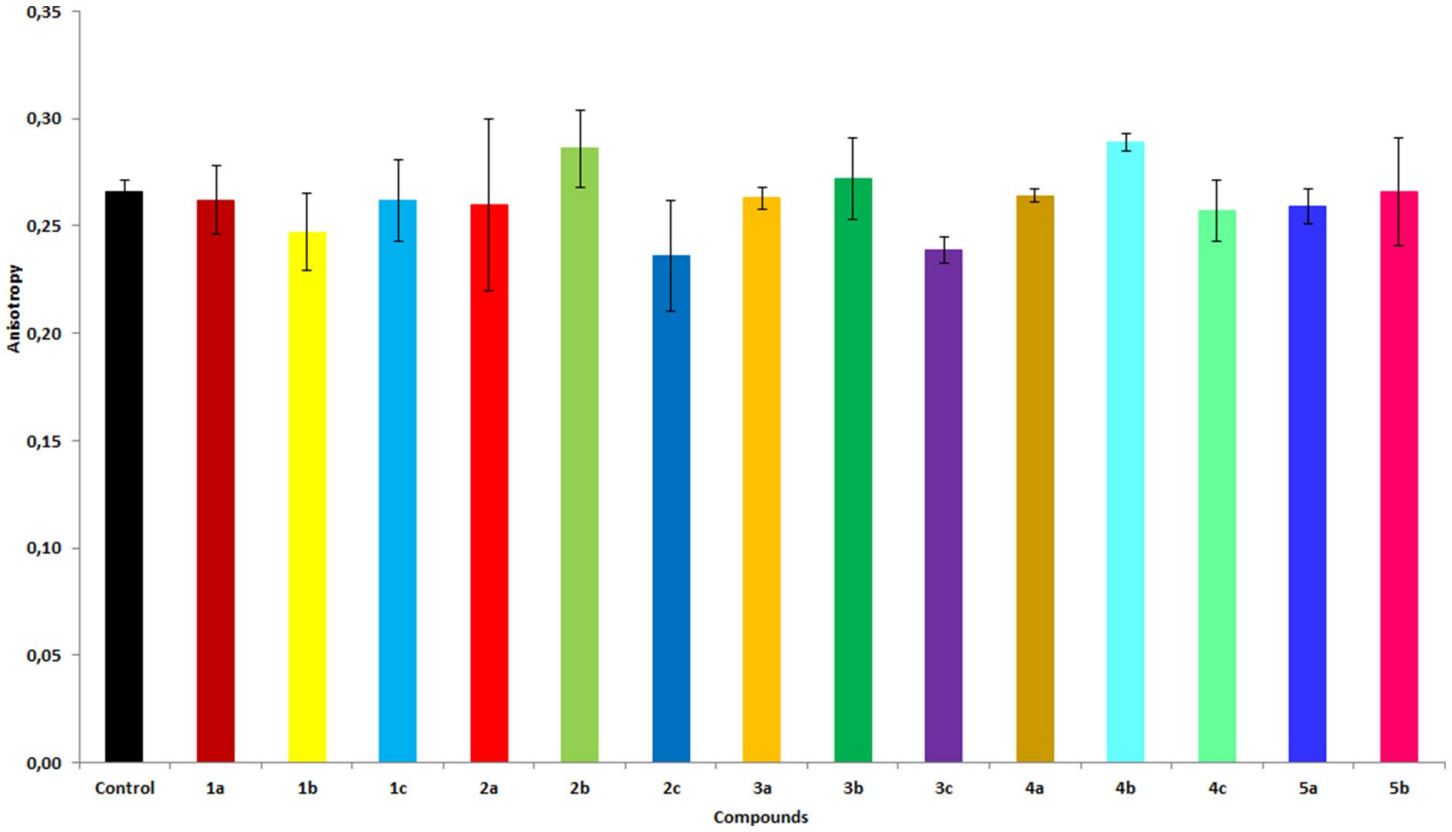
Fluorescence anisotropy values of the DPH probe in membranes of RBCs treated with the compounds at 20 μM concentration. The results are shown as mean values ± standard deviations.

The effect of each compound on the membrane fluidity of RBCs was examined using the fluorescent marker DPH. The DPH probe binds to the hydrophobic region of the membrane. Based on the change in anisotropy, one can infer the degree of change in the membrane fluidity of RBCs (Lakowicz, 2006). An increase in the anisotropy value indicates an increase in membrane stiffness, whereas a decrease suggests liquefaction of the membrane (complete data are shown in the Supplementary Table S6). In general, diols, particularly **2b** and **4b**, were found to cause an increase in anisotropy (Figure 5). Interestingly, compound **3b** caused an increase in anisotropy at lower concentrations and a decrease at higher concentrations. In contrast, hydroxy ketones **2c**, **3c**, and, to a lesser extent, **4c** caused a decrease in anisotropy, indicating an increase in membrane fluidity. The biological activity of the studied compounds likely is also related to their different effects on the cell membrane; depending on their structure, they cause an increase in membrane stiffness or membrane liquefaction. Determining the exact localization of the compounds in the membrane requires further research.

### 3.7. Proliferative activity

**Figure 6:**
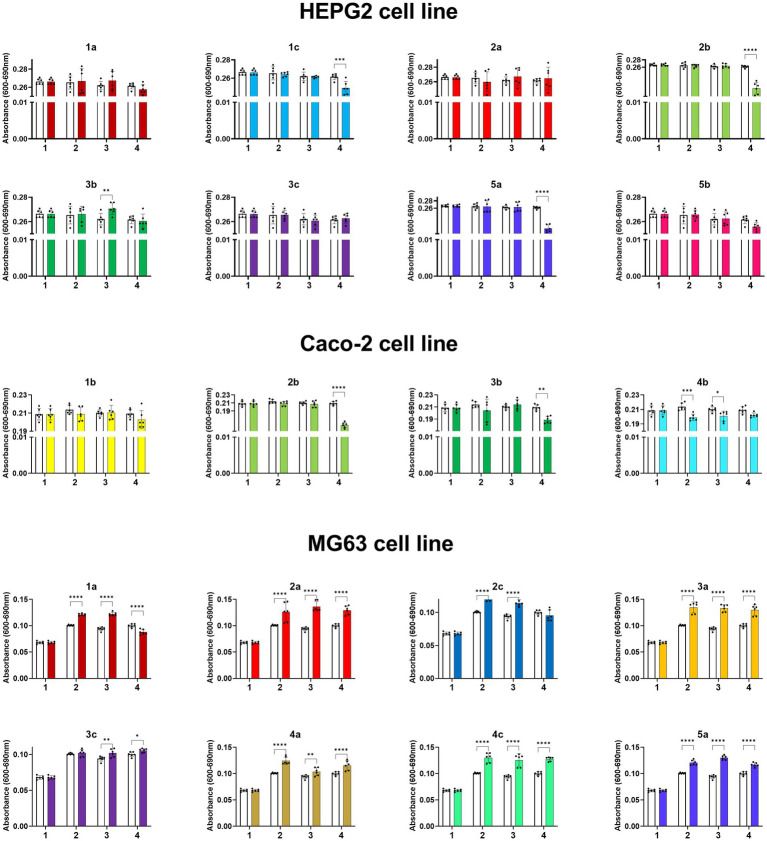
Absorbance values of HepG2, Caco-2 and MG63 cell lines treated with the compounds at concentrations from 0 to 200 mg/mL, where 1. 0 mg/mL (0% EtOH); 2. 1 mg/mL (0.01% EtOH); 3. 50 mg/mL (0.5% EtOH); 4. 200 mg/mL (2% EtOH). The results are shown as mean values ± standard deviations.

The proliferative activities of HepG2, Caco-2, and MG63 cells were differentially affected by compounds (**1a–5b**), in a dose-dependent manner (complete data are shown in the Supplementary Figures S27−S29). Compound **3b** significantly increased the proliferative potential of HepG-2 cells, whereas compound **2b** showed the highest inhibitory effect on HepG2 cell viability (Figure 6). A substantially different cellular response to the compounds was observed in the Caco-2 cell line. Caco-2 cells exhibited the lowest proliferative activity when treated with all tested compounds among all others tested cell lines. The strongest inhibition of Caco-2 cells was observed when the cells were treated with **1b**, **2b**, **3b**, and **4b**. Interestingly, **1a** and **2a** more strongly significantly suppressed the viability of HepG-2 cells. Compound **5b** at 1 μg/mL induced the proliferative activity of all cell lines to a similar level as compound **3c** did at 50 μg/mL. The proliferation of MG63 cells was differentially modulated by the compounds, depending on the dosage. Compounds **1c**, **3b**, and **5b** suppressed the proliferation of MG63 cells. However, at 50 μg/mL, **5b** significantly increased cell viability. Interestingly, compounds **1a**, **2a**, **2c**, **3a**, **3c**, **4a, 4c**, and **5a** promoted MGC63 proliferation.

The results obtained by us for starting propenylbenzenes **1-5a** are in agreement with the available literature. Anethol (**3a**), safrole and eugenol (isomers of compounds **1a** and **5a**) inhibited the growth of human hepatocellular carcinoma HepG2 cells (Yoo et al., 2005; Song et al., 2014, 2020). Whereas, isosafrole (**1a**) and eugenol induced apoptosis in human colon carcinoma Caco-2 cell line (Lea et al., 2016; Padhy et al., 2022). In human osteosarcoma MG63 cell line, *trans*-anethole (**3a**) caused abrogation in proliferation and induces apoptosis through the mitochondrial mediated pathway (Pandit et al., 2020). The effect of compounds **1-5b** and **1-4c** on the cell lines we studied has not been described in the available literature so far.

## 4. Conclusion

A two-step chemo-enzymatic method for obtaining oxygenated derivatives of commercially available propenylbenzenes was developed. The process involves chemo-enzymatic epoxidation followed by epoxide hydrolysis of starting compounds **1a–5a** to corresponding diols **1b–5b**, followed by microbial oxidation of thus obtained diols **1b–5b** into hydroxy ketones **1c–4c**. Among bacteria from different genera, *Dietzia* sp. DSM44016, *R. erythropolis* DSM44534, *R. erythropolis* PCM2150, and *R. ruber* PCM2166 effectively oxidized diols **1b–4b** to corresponding hydroxy ketones **1c–4c**, indicating high alcohol dehydrogenase activity. Bio-oxidation performed on preparative scale afforded hydroxy ketones **1c–4c** with good isolation yields. The obtained compounds and starting propenylbenzenes were tested for antimicrobial, antioxidant, haemolytic, and anticancer activities, and their impact on membrane fluidity. Compounds **1a**, **3a–c**, **4a,b**, and **5a,b** showed high fungistatic activity against selected strains of *C. albicans*. The type of substituent can significantly affect the MIC_50_ value. The starting propenylbenzenes **1a–5a** with a double bond in their structure showed the highest antiradical activity among the tested compounds. The compounds showed no cytotoxicity against human RBCs. However, compounds **2b–4b** and **2c–4c** affected the fluidity of the RBCs membrane; diols generally increased membrane stiffness, whereas hydroxy ketones increased membrane fluidity. Several compounds inhibited or promoted HepG2, Caco-2, and MG63 cell proliferation, depending on the concentration they were used at. Obtained data shed promising light on the application of tested compounds as hepatoprotective and promoting bone remodeling.

## Data availability statement

The original contributions presented in the study are included in the article/Supplementary material, further inquiries can be directed to the corresponding authors.

## Author contributions

DH, FB, and EB: conceptualization. DH, AW, HP, KM, and FB: formal analysis. DH: funding acquisition. DH, ES, MG, AW, HP, and MM: investigation. DH, AW, HP, KM, TO, EB, and FB: methodology. DH and FB: resources. FB and EB: supervision. DH, AW, HP, and KM: visualization. DH, AW, HP, KM, and FB: writing – original draft. ES, TO, and EB: writing – review and editing. All authors contributed to the article and approved the submitted version.

## Funding

This research was funded by the project “UPWR 2.0: international and interdisciplinary program of development of Wrocław University of Environmental and Life Sciences,” co-financed by the European Social Fund under the Operational Program Knowledge Education Development, under contract No. POWR.03.05.00-00-Z062/18 of 4 June 2019. The APC is financed by Wroclaw University of Environmental and Life Science.

## Conflict of interest

The authors declare that the research was conducted in the absence of any commercial or financial relationships that could be construed as a potential conflict of interest.

## Publisher’s note

All claims expressed in this article are solely those of the authors and do not necessarily represent those of their affiliated organizations, or those of the publisher, the editors and the reviewers. Any product that may be evaluated in this article, or claim that may be made by its manufacturer, is not guaranteed or endorsed by the publisher.
