# Supplementary material for: Chemo-enzymatic synthesis and biological activity evaluation of propenylbenzene derivatives
Source: Front Microbiol. 2023 Jun 26;14:1223123. doi: 10.3389/fmicb.2023.1223123 (PMC10330721; doi:10.3389/fmicb.2023.1223123)
Supplement: Supplementary file 1 [file Data_Sheet_1.PDF]

Supplementary Material

Chemo-enzymatic Synthesis and Biological Activity Evaluation of Propenylbenzene Derivatives

Dawid Hernik[1*], Ewa Szczepańska[1], Maria Chiara Ghezzi[2], Elisabetta Brenna[2], Aleksandra Włoch[3], Hanna Pruchnik[3], Malwina Mularczyk[4], Krzysztof Marycz[4], Teresa Olejniczak[1] and Filip Boratyński[1*]

[1]Department of Food Chemistry and Biocatalysis, Wroclaw University of Environmental and Life Sciences, Wrocław, Poland

[2]Dipartimento di Chimica, Materiali ed Ingegneria Chimica "*Giulio Natta*", Politecnico di Milano, Milano, Italy

[3]Department of Physics and Biophysics, Wroclaw University of Environmental and Life Sciences, Wrocław, Poland

[4]Department of Experimental Biology, Wroclaw University of Environmental and Life Sciences, Wrocław, Poland

*** Correspondence:** Corresponding Authors:

dawid.hernik@upwr.edu.pl; filip.boratynski@upwr.edu.pl

Number of pages: 27
Number of Figures: 29
Number of Tables: 6

1. Supplementary Data

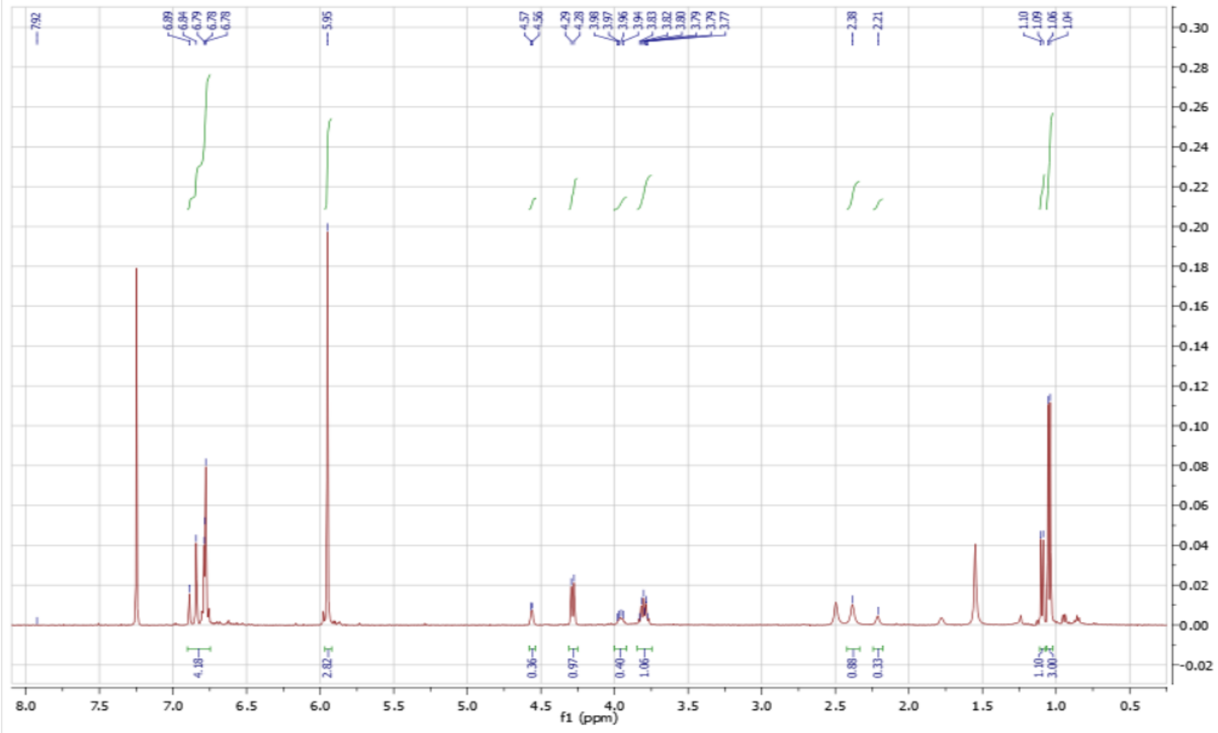

Figure S1. ^{1}H NMR spectrum of (1*R**,2*S**) and (1*R**,2*R**)-1-(1,3-benzodioxol-5-yl)propane-1,2-diol (**1b**).

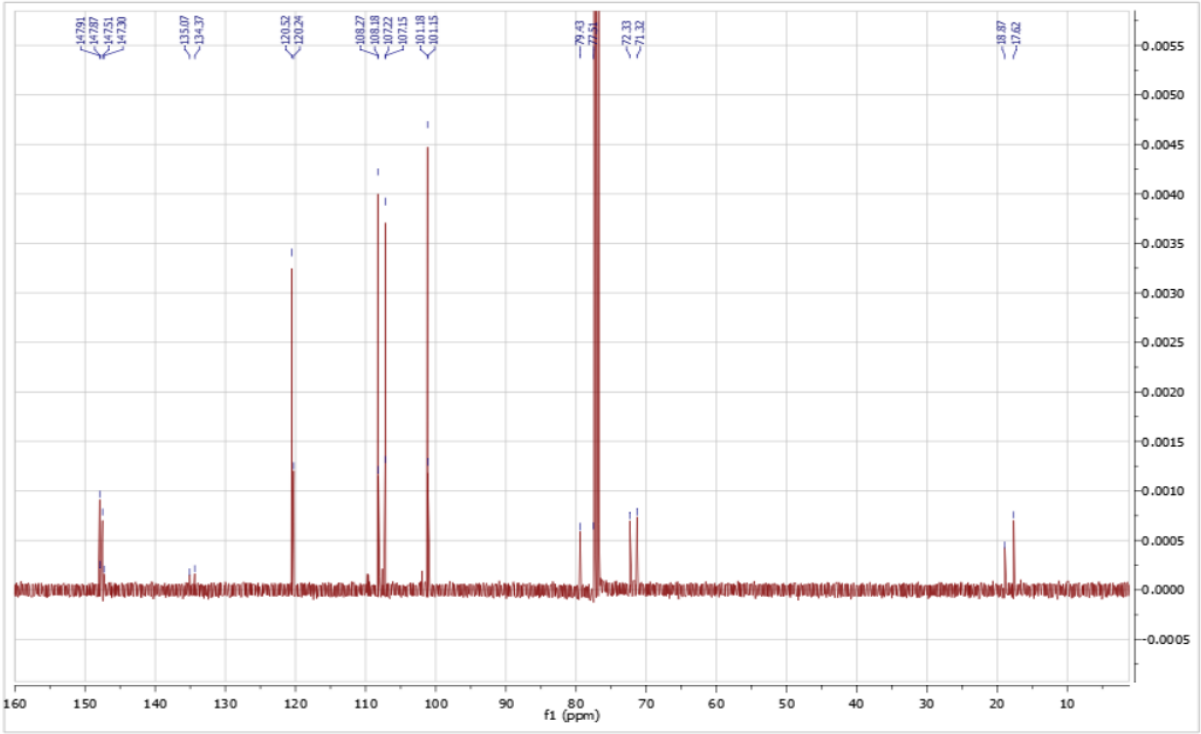

Figure S2. ^{13}C NMR spectrum of (1*R**,2*S**) and (1*R**,2*R**)-1-(1,3-benzodioxol-5-yl)propane-1,2-diol (**1b**).

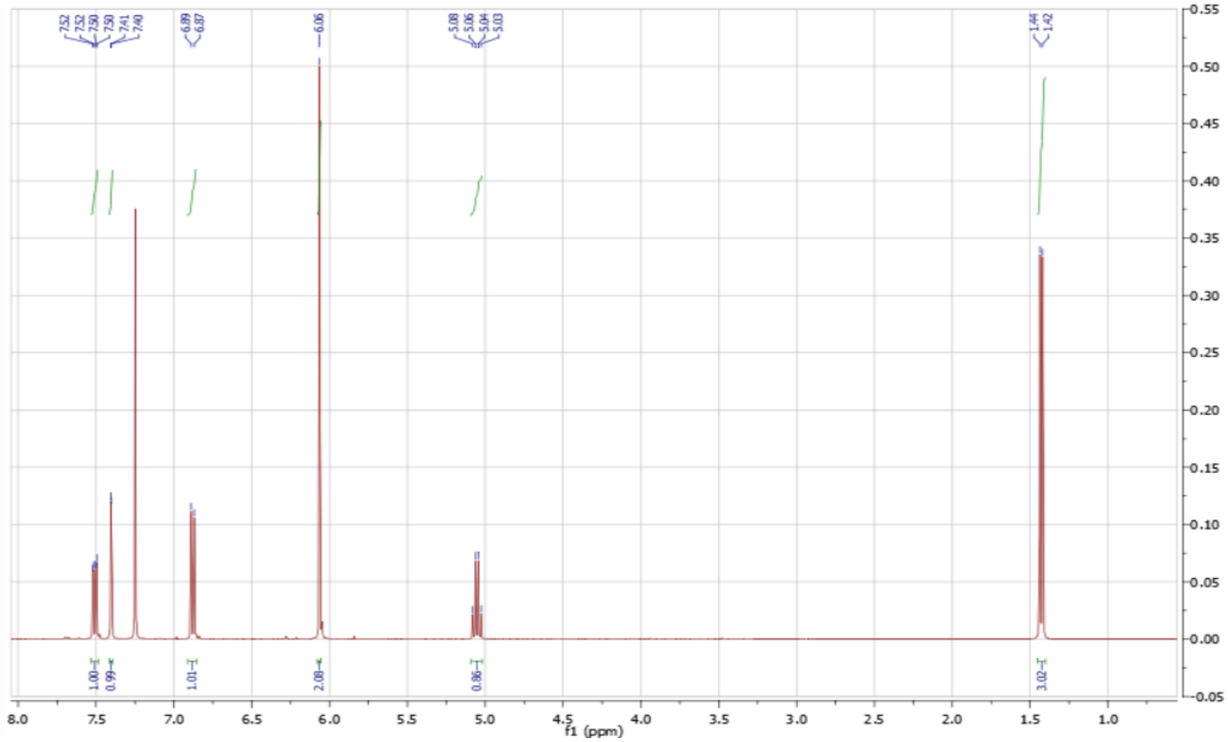

Figure S3. ^{1}H NMR spectrum of 1-(1,3-benzodioxol-5-yl)-2-hydroxypropan-1-one (**1c**).

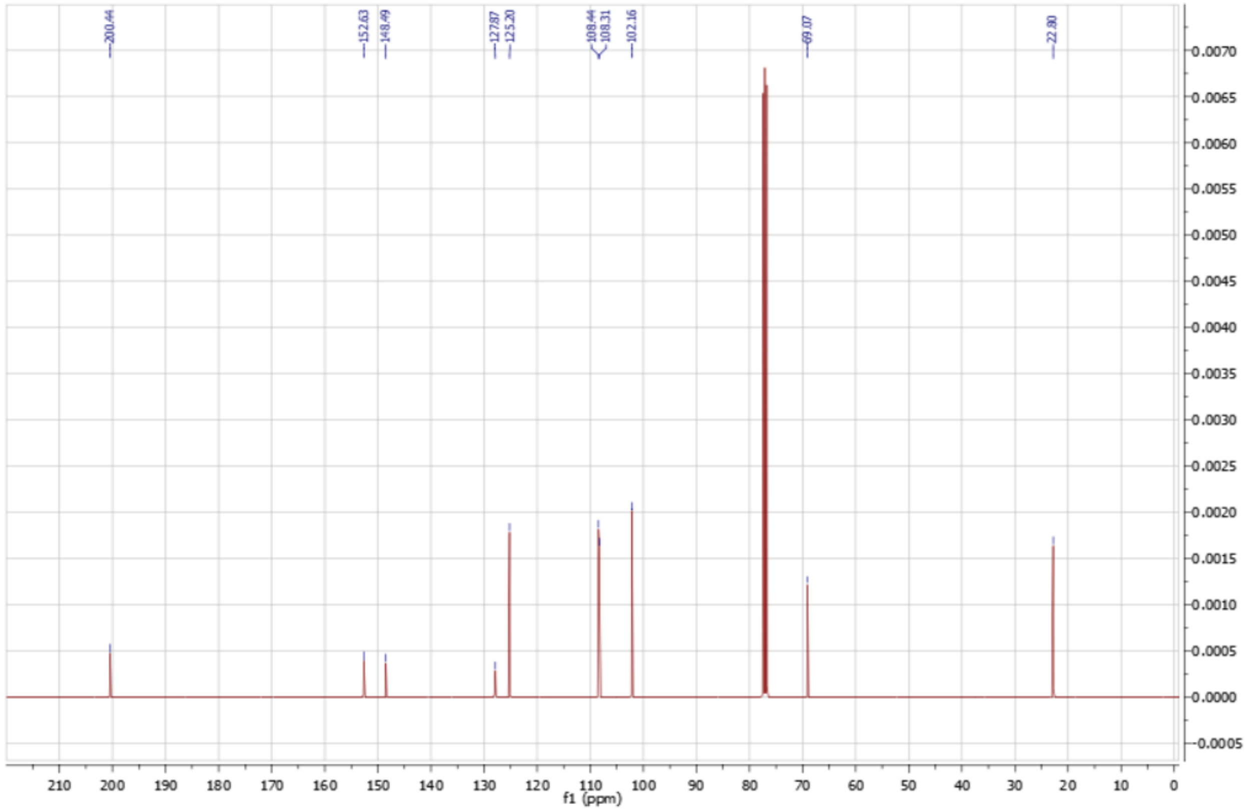

Figure S4. ^{13}C NMR spectrum of 1-(1,3-benzodioxol-5-yl)-2-hydroxypropan-1-one (**1c**).

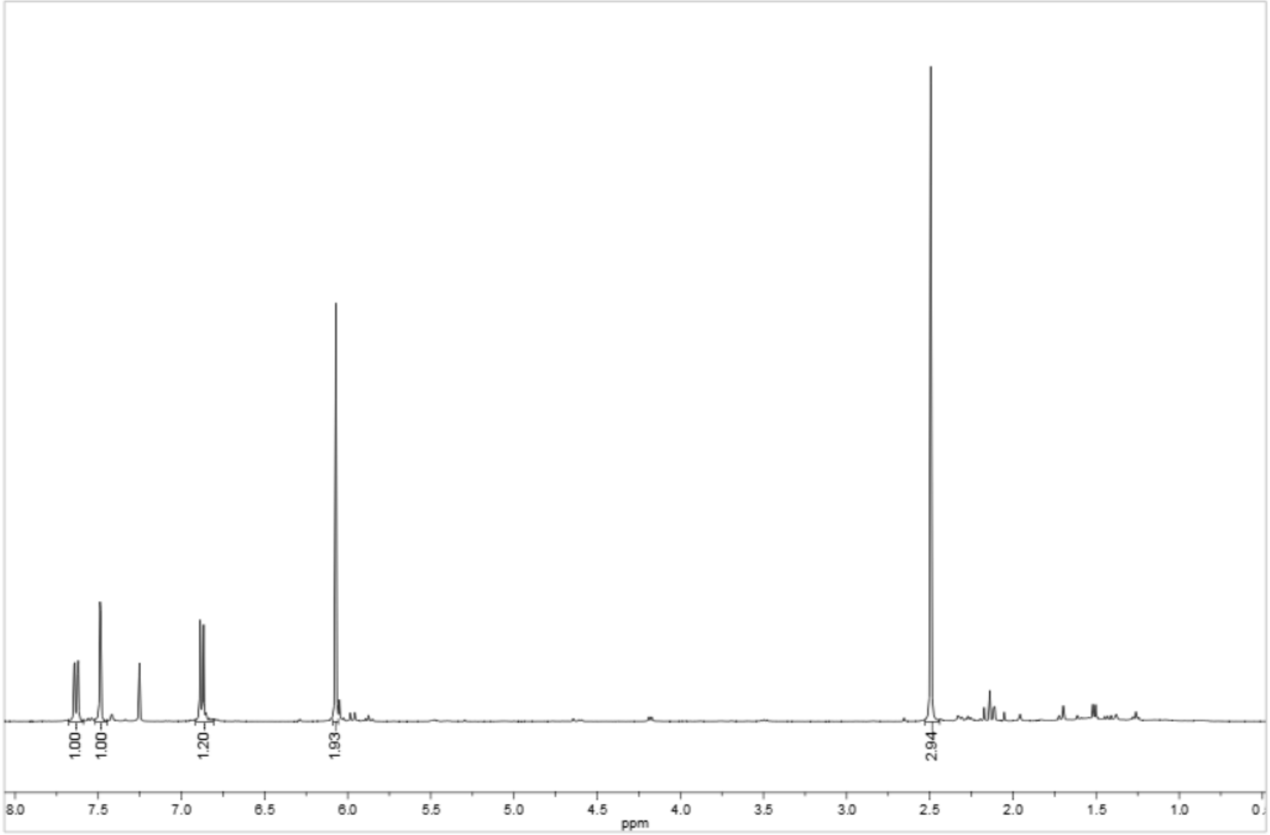

Figure S5. ^{1}H NMR spectrum of 1-(1,3-benzodioxol-5-yl)propane-1,2-dione.

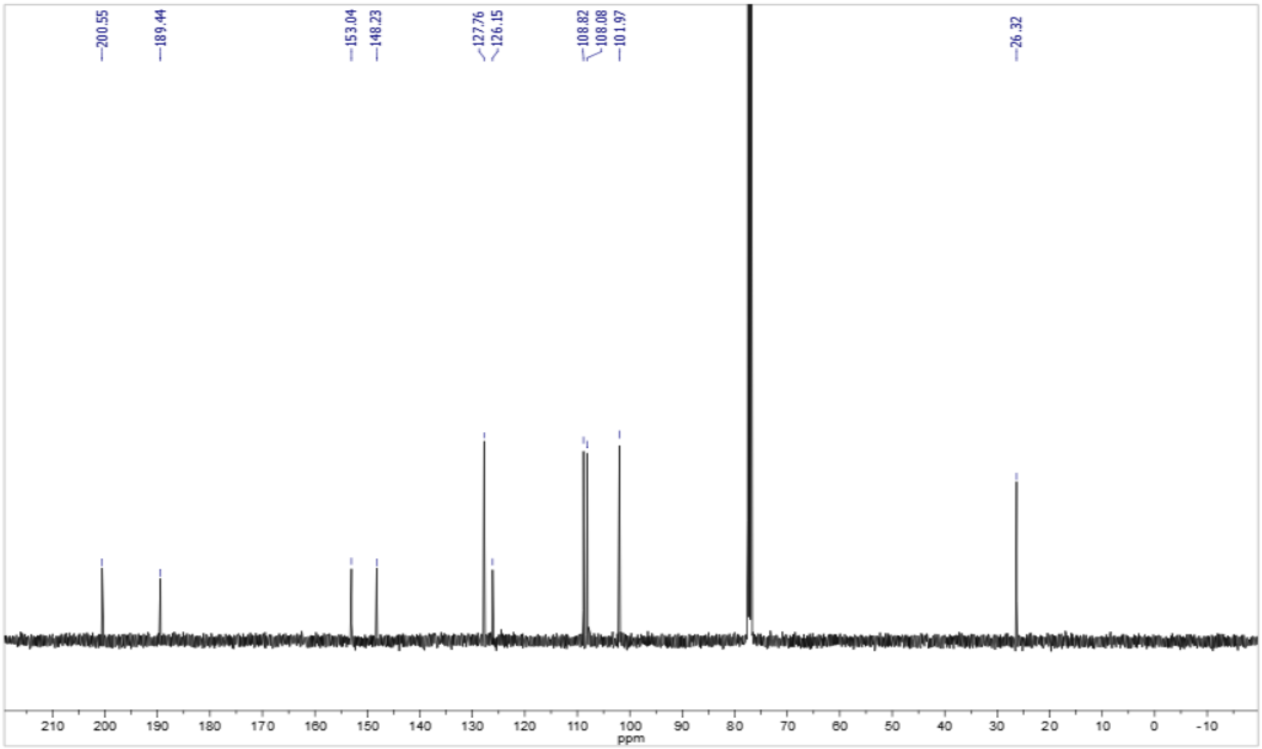

Figure S6. ^{13}C NMR spectrum of 1-(1,3-benzodioxol-5-yl)propane-1,2-dione.

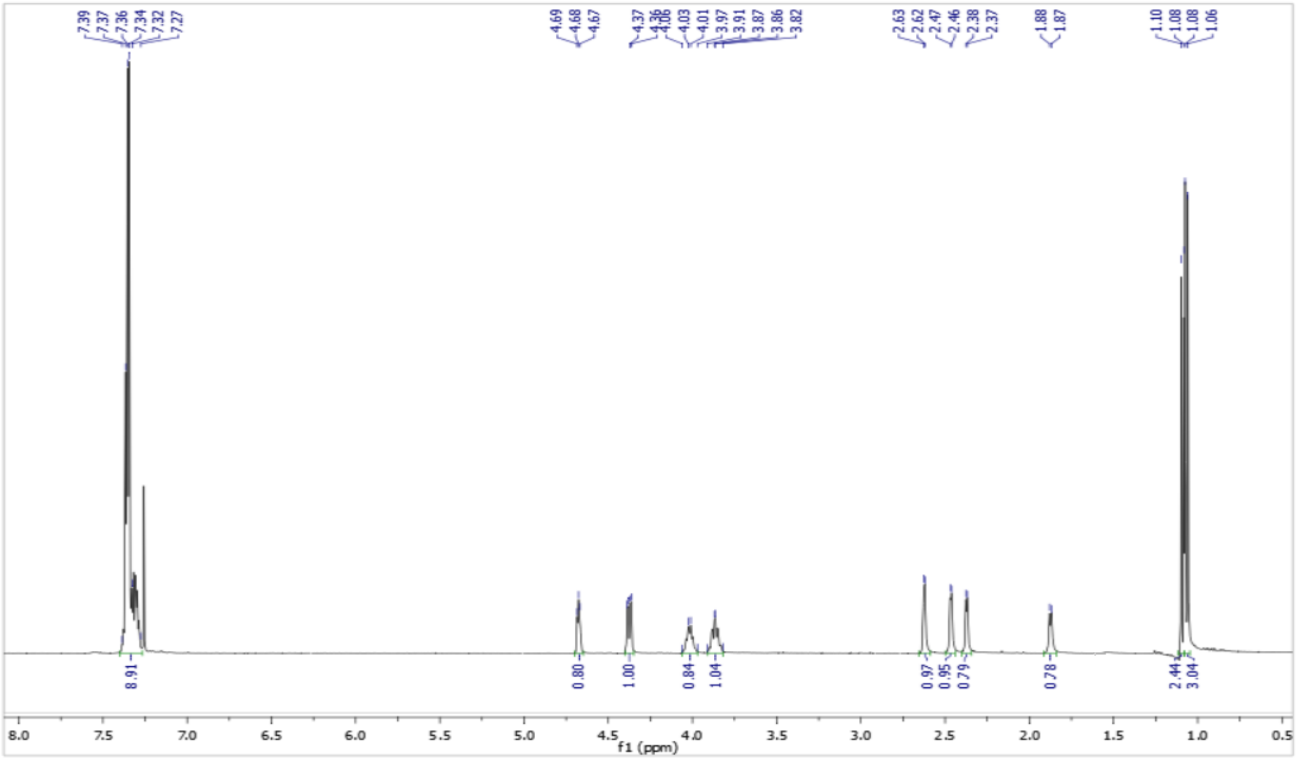

Figure S7. ^{1}H NMR spectrum of (1*R**,2*S**) and (1*R**,2*R**)-1-phenylpropane-1,2-diol (**2b**).

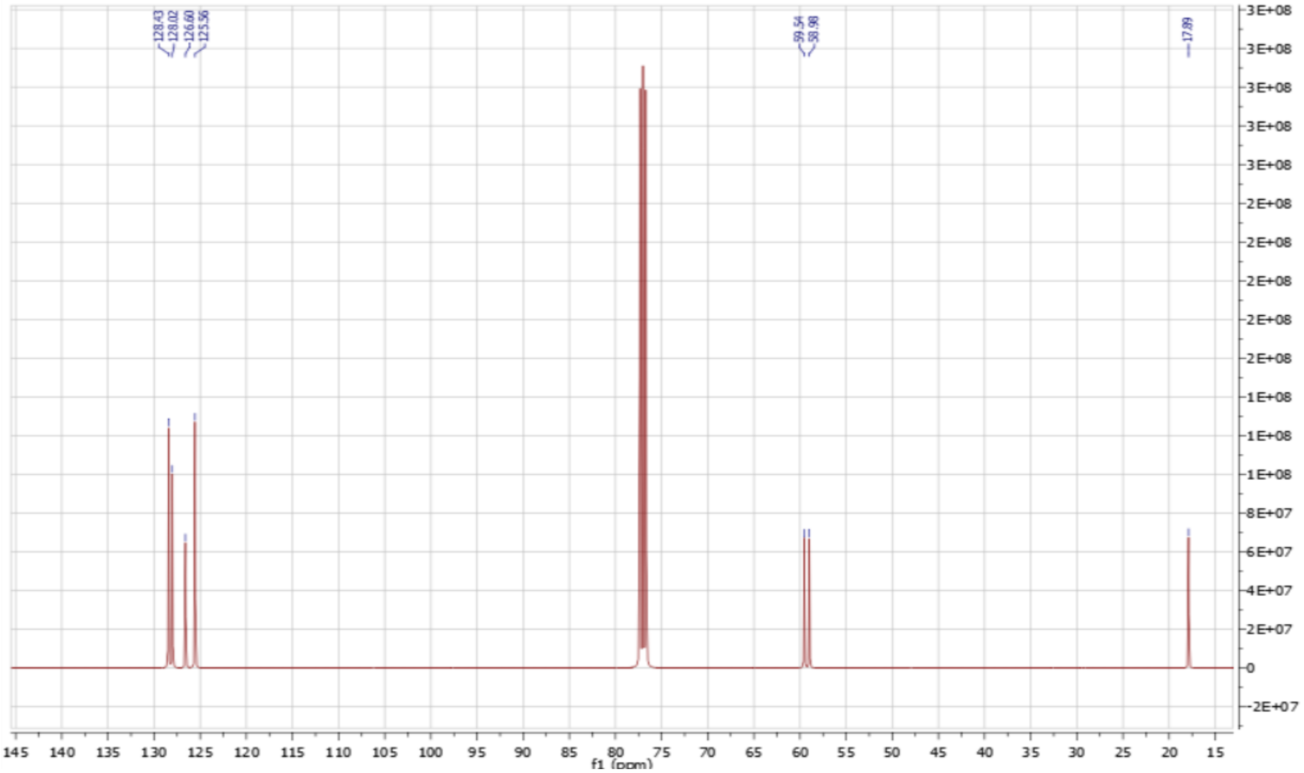

Figure S8. ^{13}C NMR spectrum of (1*R**,2*S**) and (1*R**,2*R**)-1-phenylpropane-1,2-diol (**2b**).

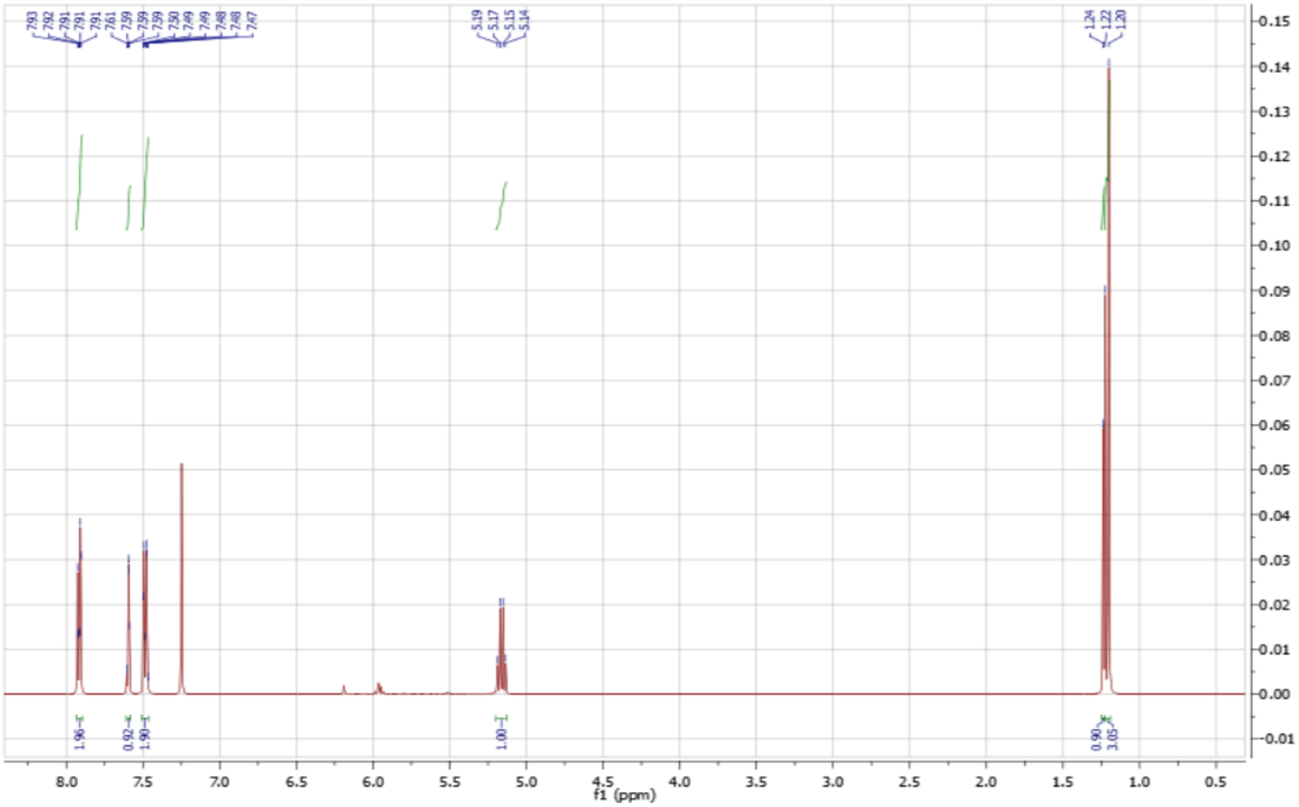

Figure S9. ^{1}H NMR spectrum of 2-hydroxy-1-phenylpropan-1-one (**2c**).

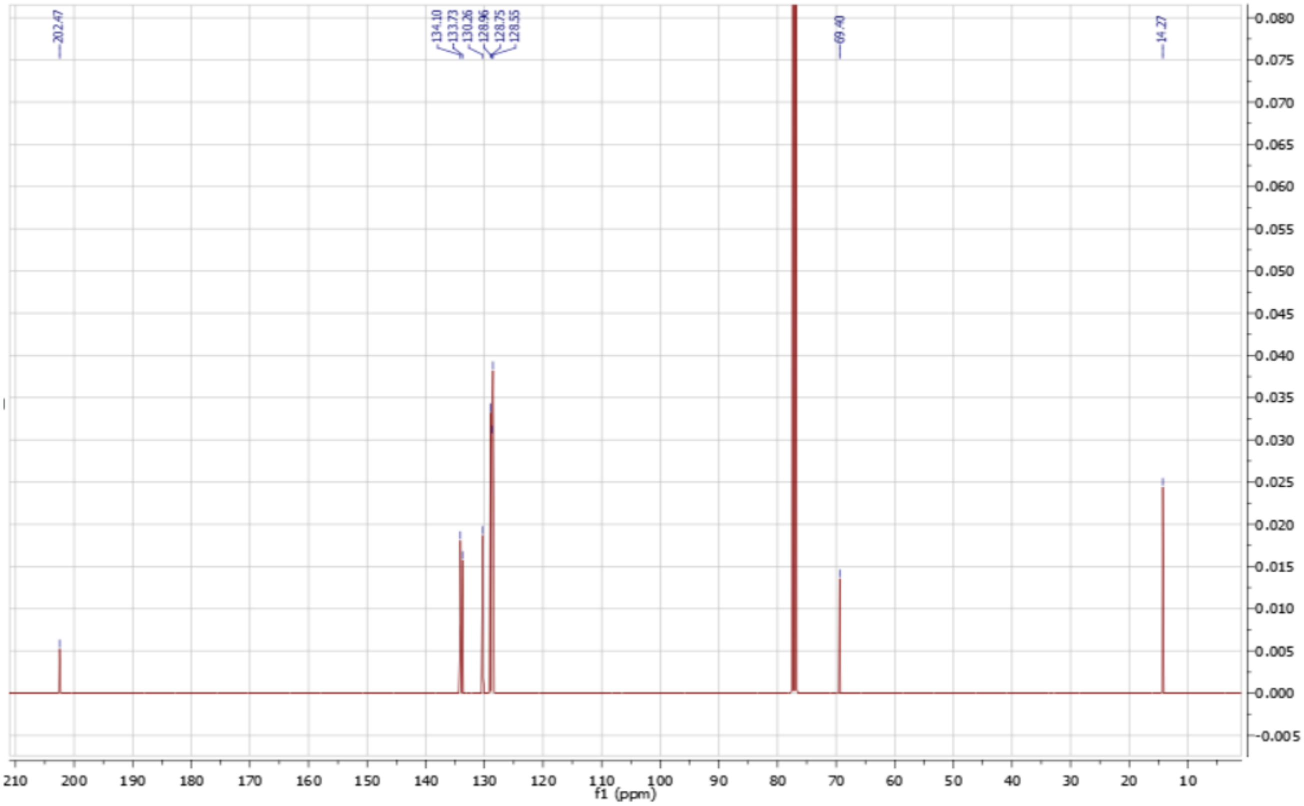

Figure S10. ^{13}C NMR spectrum of 2-hydroxy-1-phenylpropan-1-one (**2c**).

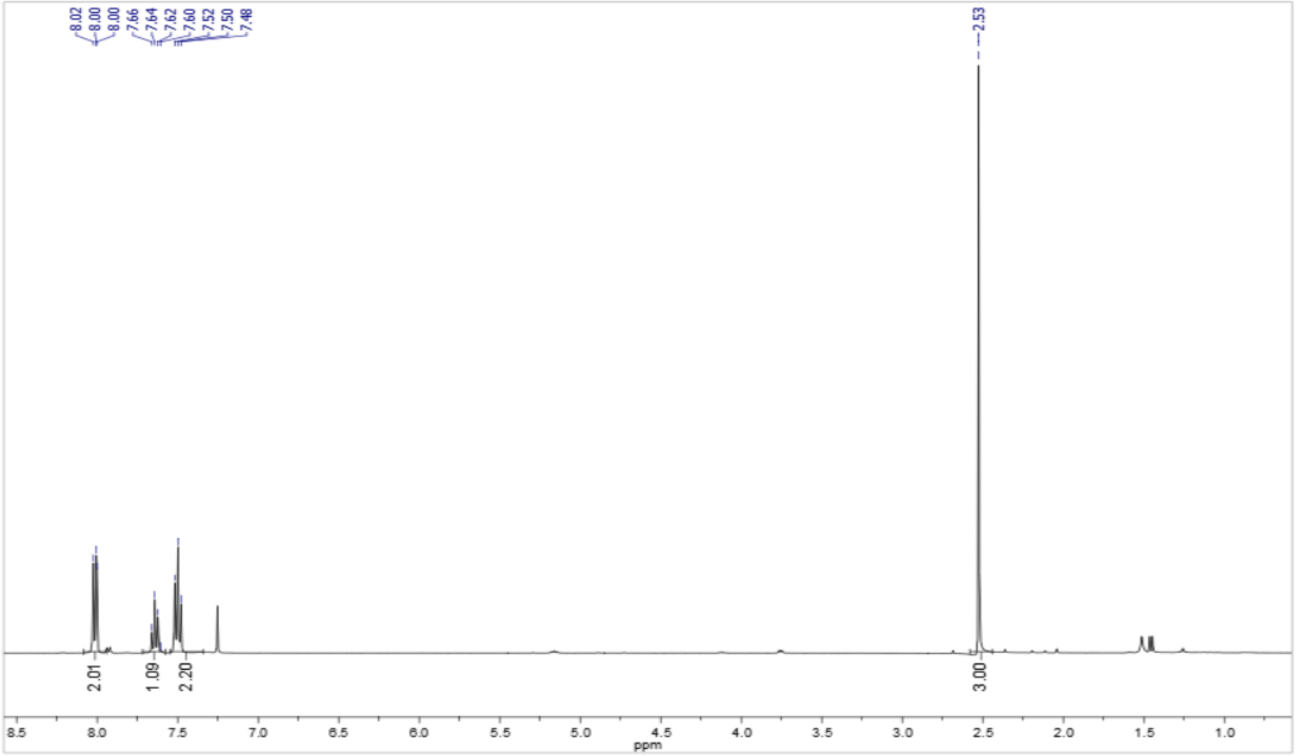

Figure S11. ^{1}H NMR spectrum of 1-phenylpropane-1,2-dione.

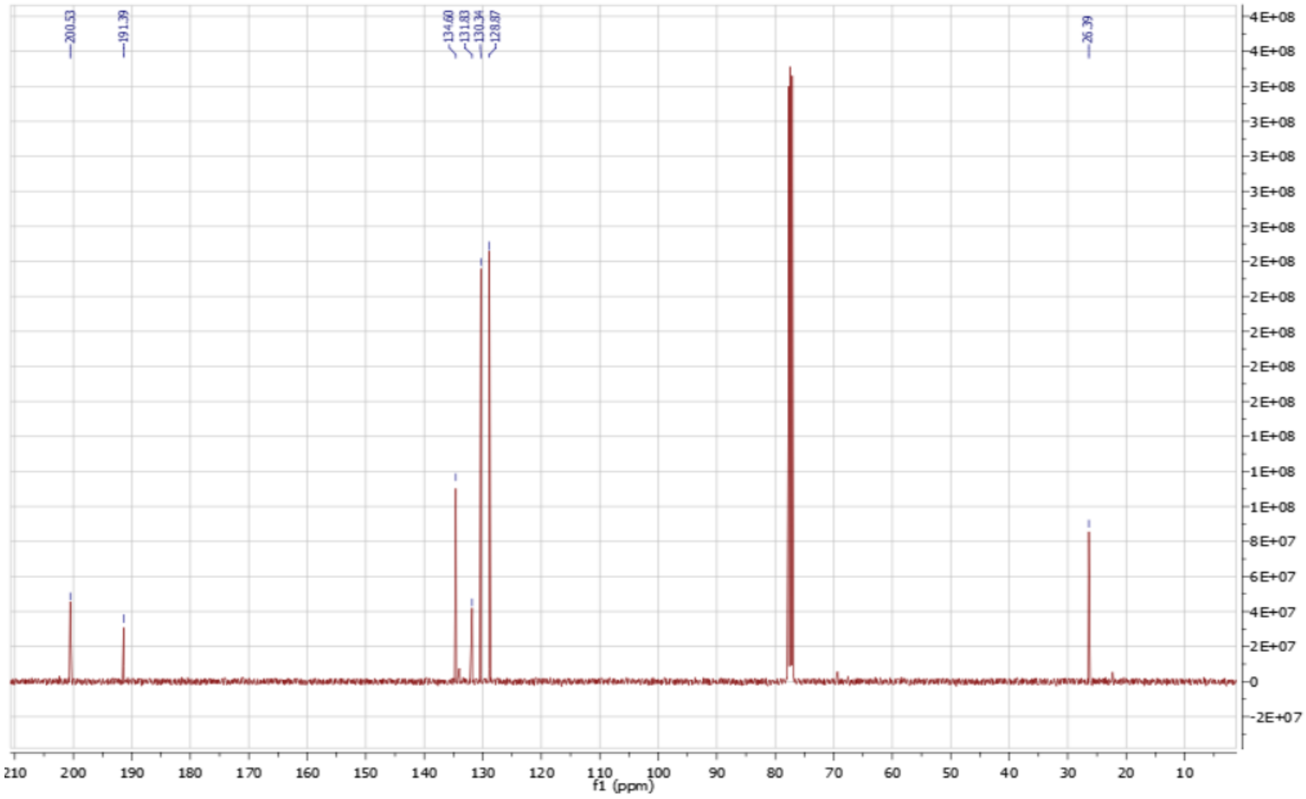

Figure S12. ^{13}C NMR spectrum of 1-phenylpropane-1,2-dione.

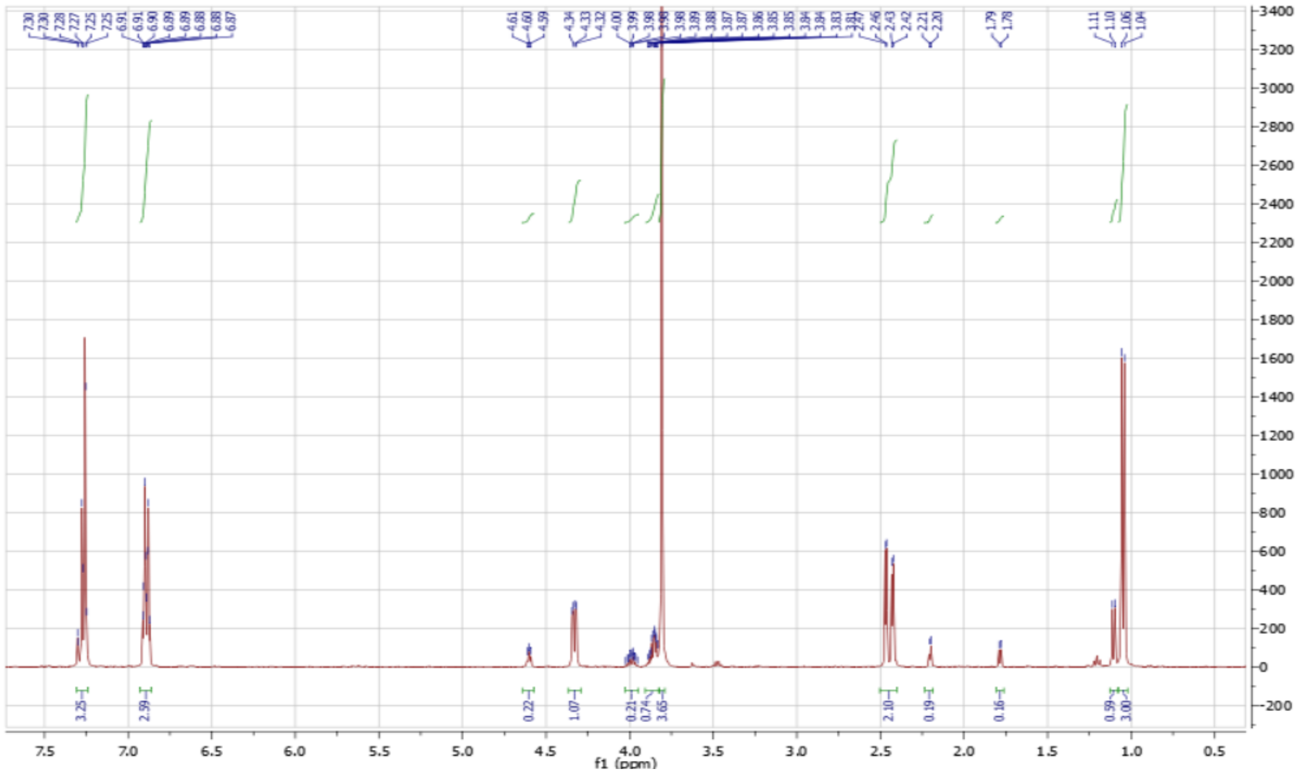

Figure S13. ^{1}H NMR spectrum of (1*R**,2*S**) and (1*R**,2*R**)-1-(4-methoxyphenyl)propane-1,2-diol (**3b**).

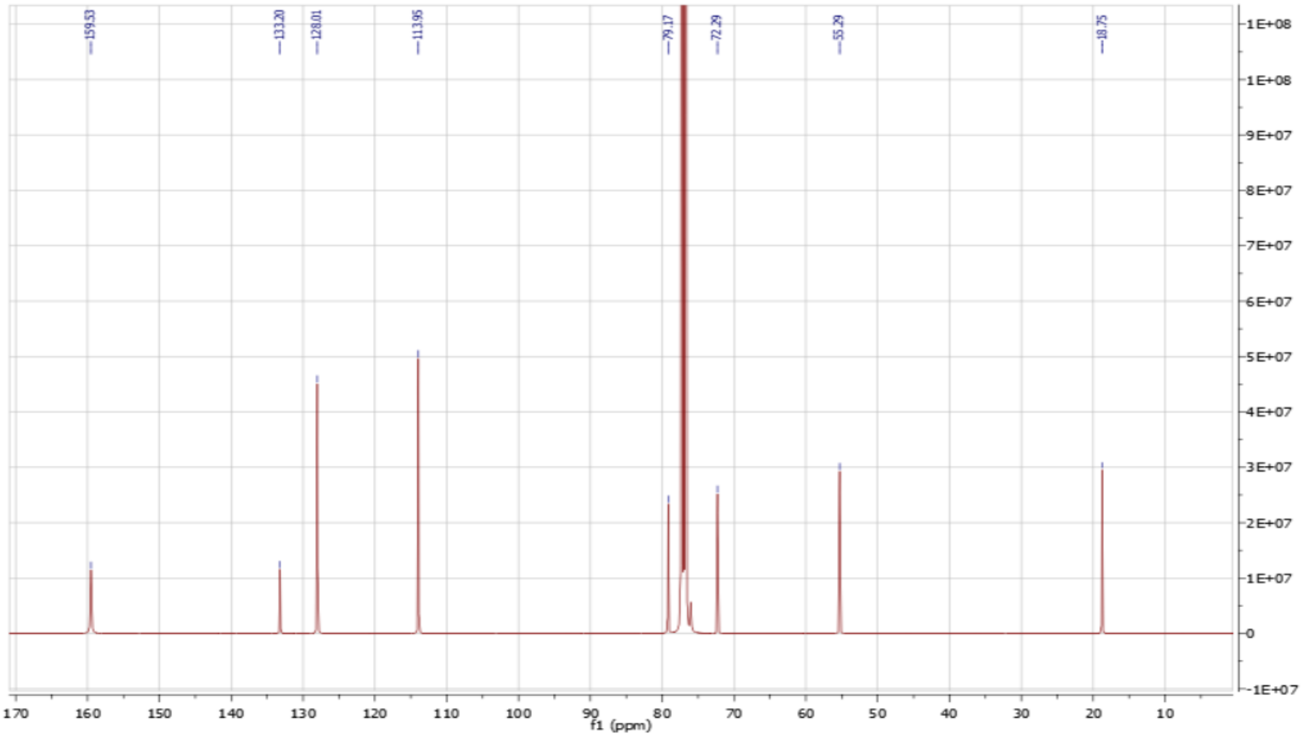

Figure S14. ^{13}C NMR spectrum of (1*R**,2*S**) and (1*R**,2*R**)-1-(4-methoxyphenyl)propane-1,2-diol (**3b**).

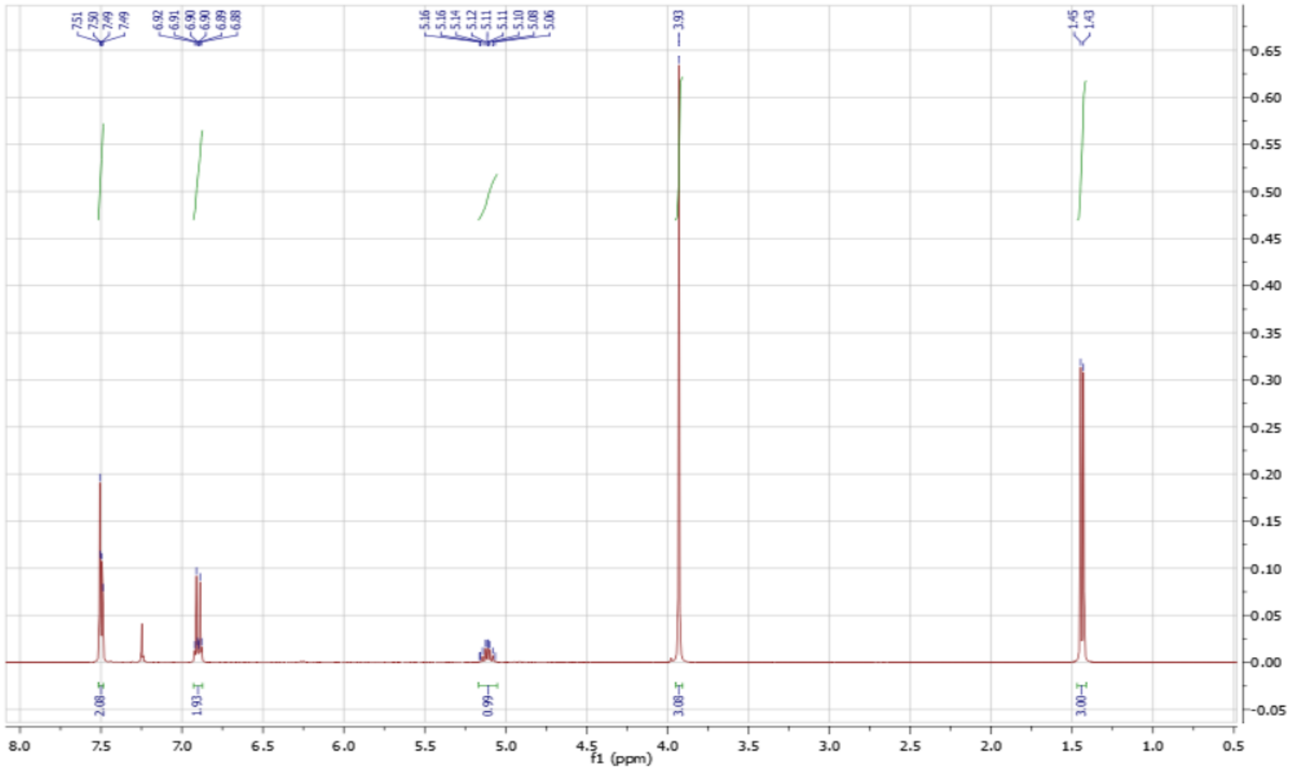

Figure S15. ^{1}H NMR spectrum of 2-hydroxy-1-(4-methoxyphenyl)propan-1-one (**3c**).

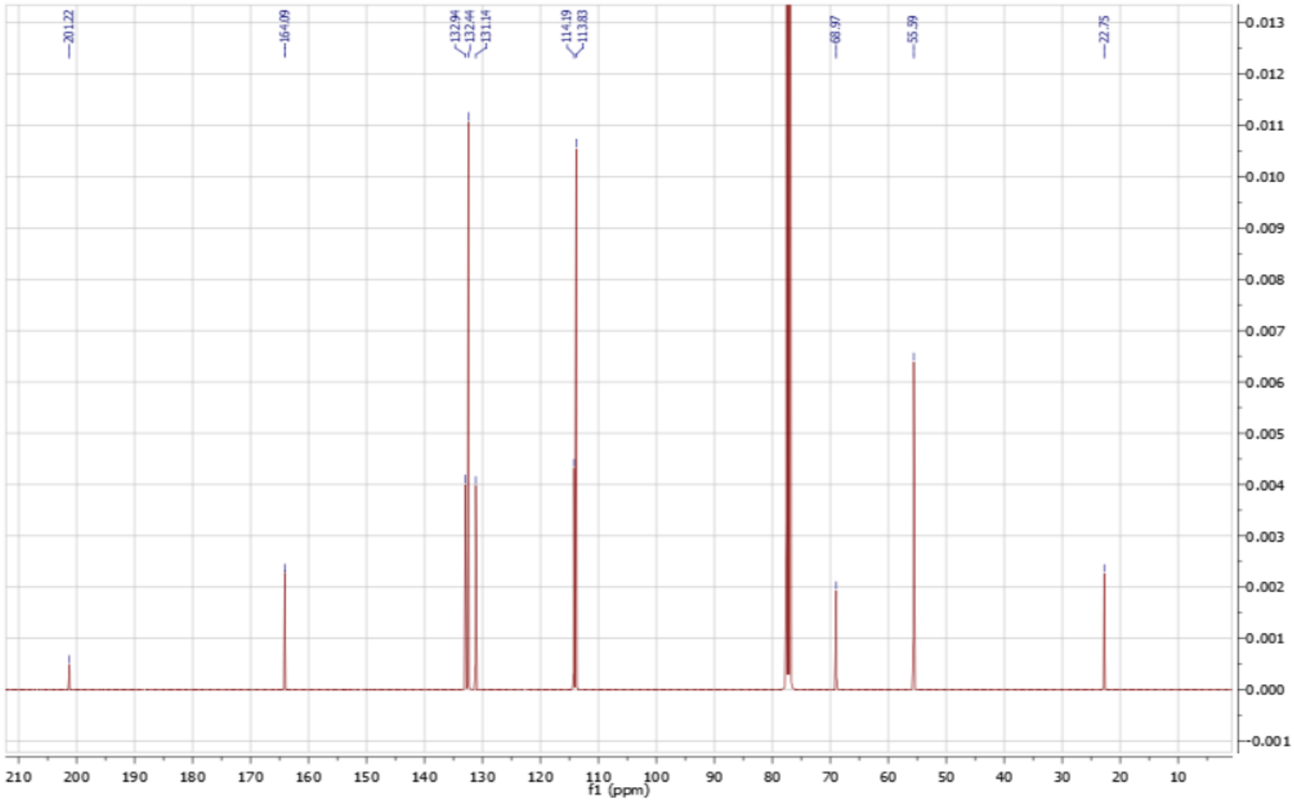

Figure S16. ^{13}C NMR spectrum of 2-hydroxy-1-(4-methoxyphenyl)propan-1-one (**3c**).

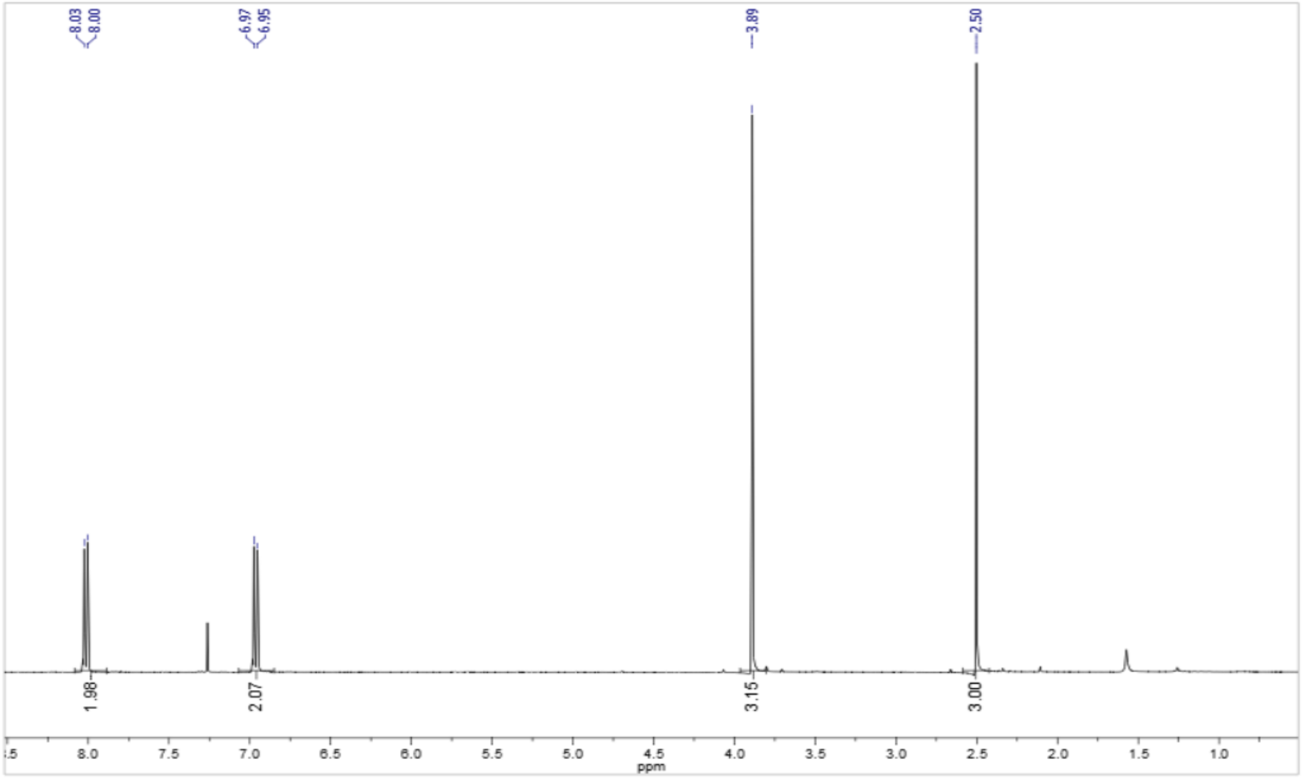

Figure S17. ^{1}H NMR spectrum of 1-(4-methoxyphenyl)propane-1,2-dione.

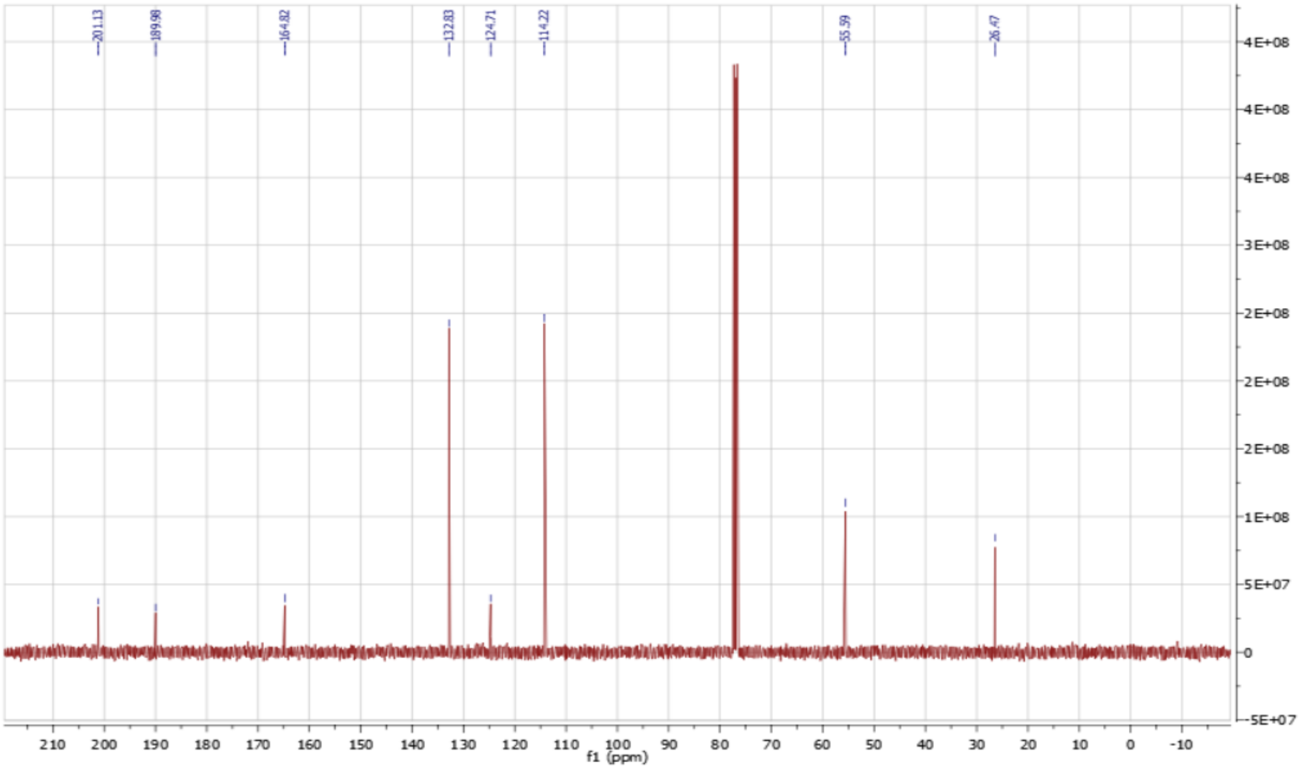

Figure S18. ^{13}C NMR spectrum of 1-(4-methoxyphenyl)propane-1,2-dione.

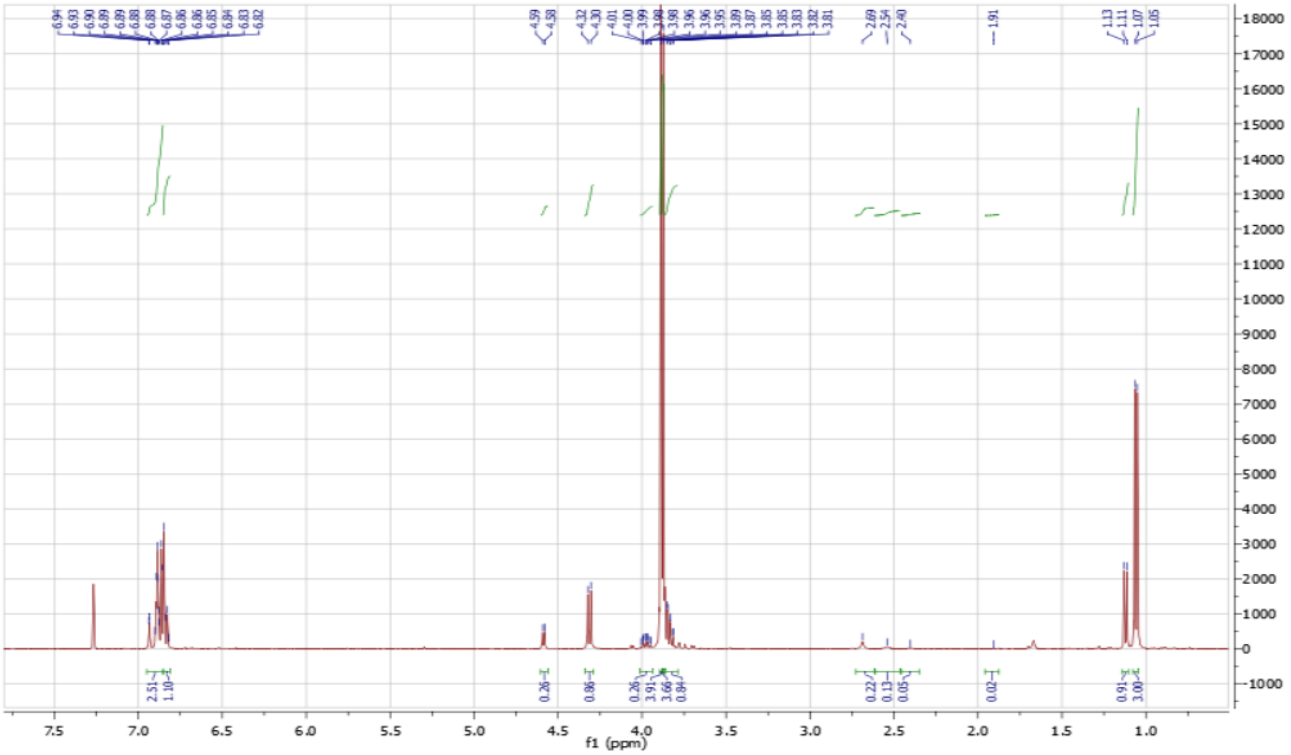

Figure S19. ^{1}H NMR spectrum of (1*R**,2*S**) and (1*R**,2*R**)-1-(3,4-dimethoxyphenyl)propane-1,2-diol (**4b**).

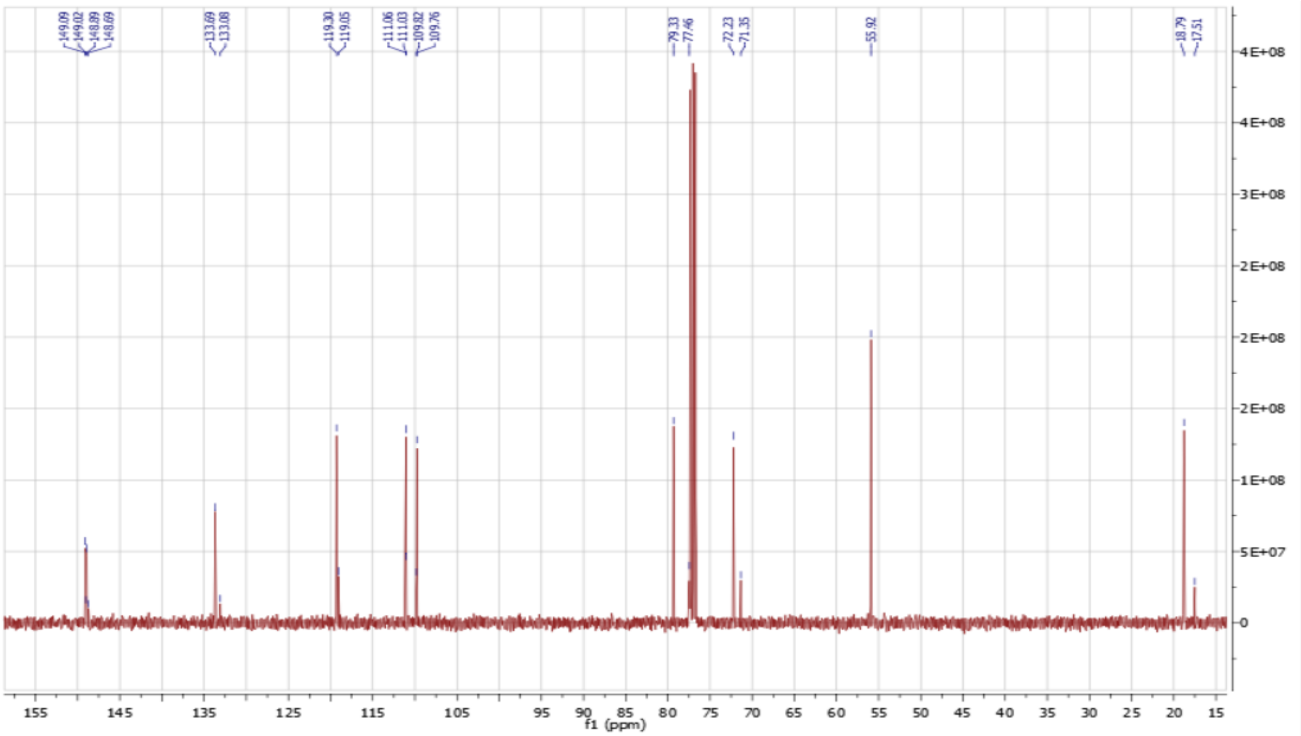

Figure S20. ^{13}C NMR spectrum of (1*R**,2*S**) and (1*R**,2*R**)-1-(3,4-dimethoxyphenyl)propane-1,2-diol (**4b**).

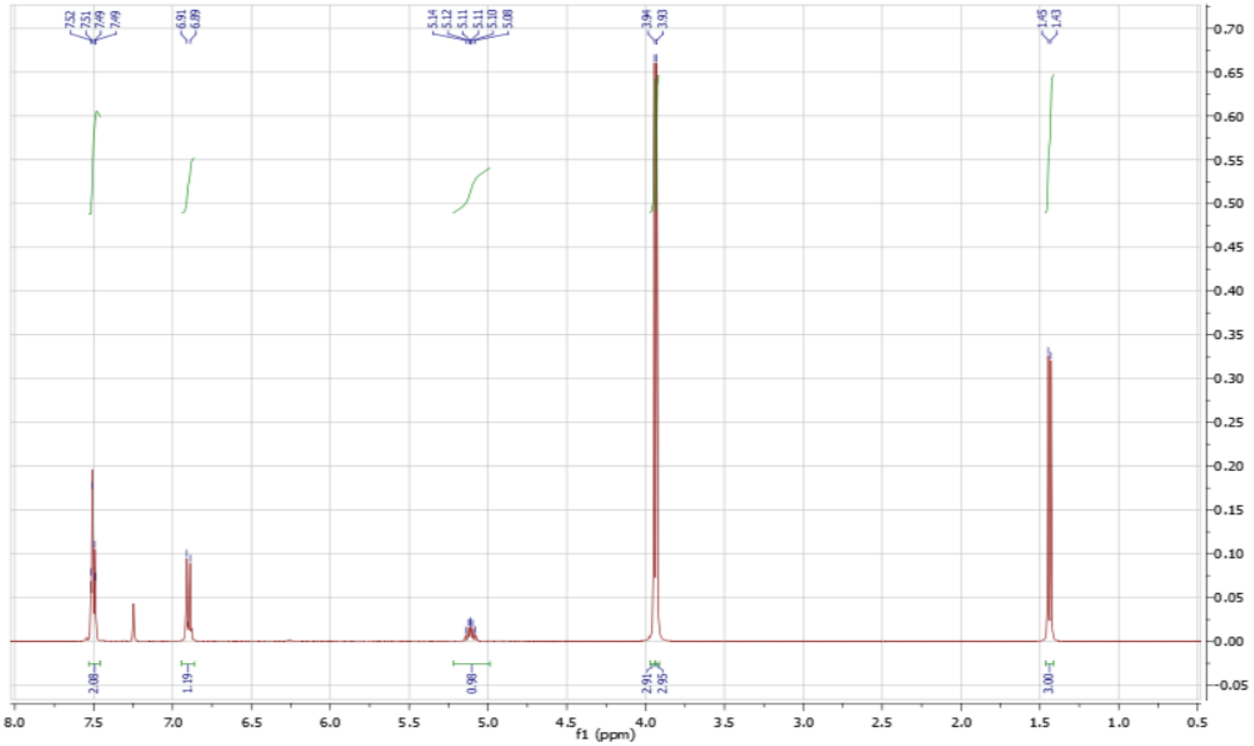

Figure S21. ^{1}H NMR spectrum of 1-(3,4-dimethoxyphenyl)-2-hydroxypropan-1-one (**4c**).

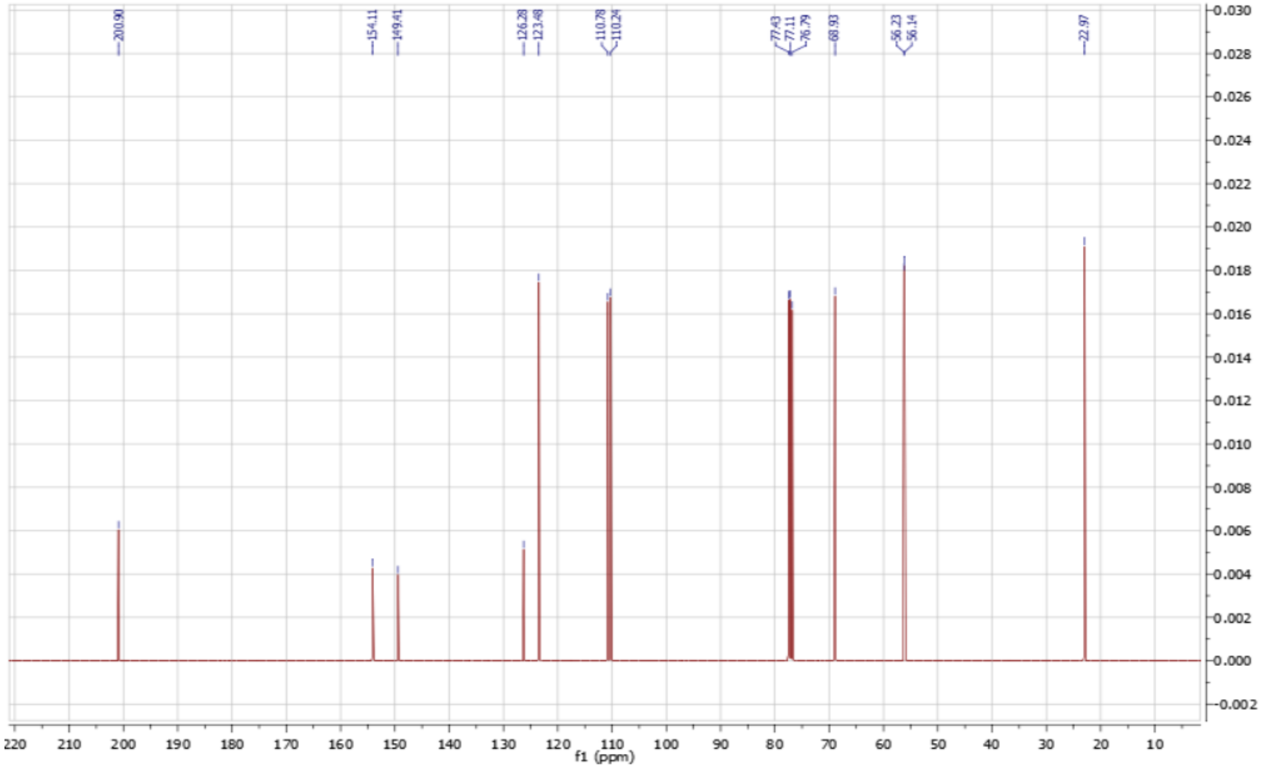

Figure S22. ^{13}C NMR spectrum of 1-(3,4-dimethoxyphenyl)-2-hydroxypropan-1-one (**4c**).

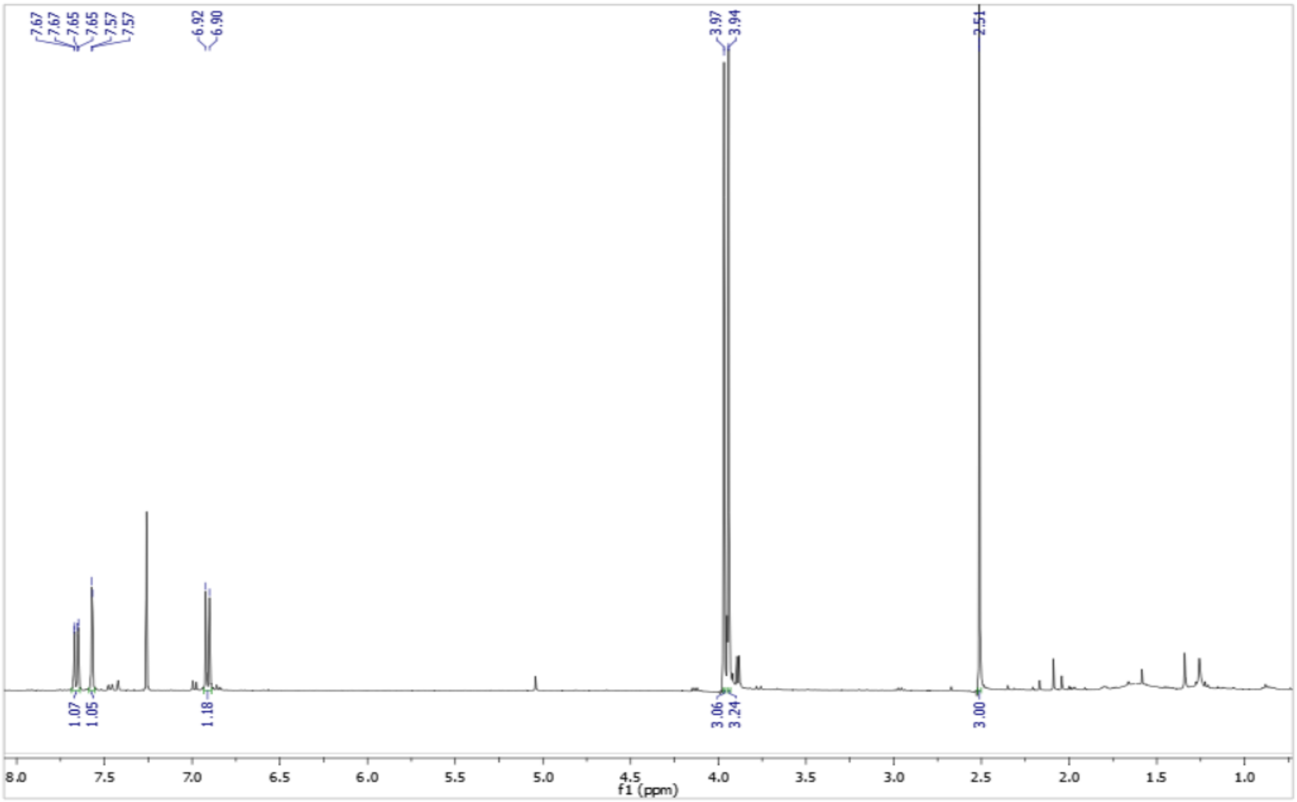

Figure S23. ^{1}H NMR spectrum of 1-(3,4-dimethoxyphenyl)propane-1,2-dione.

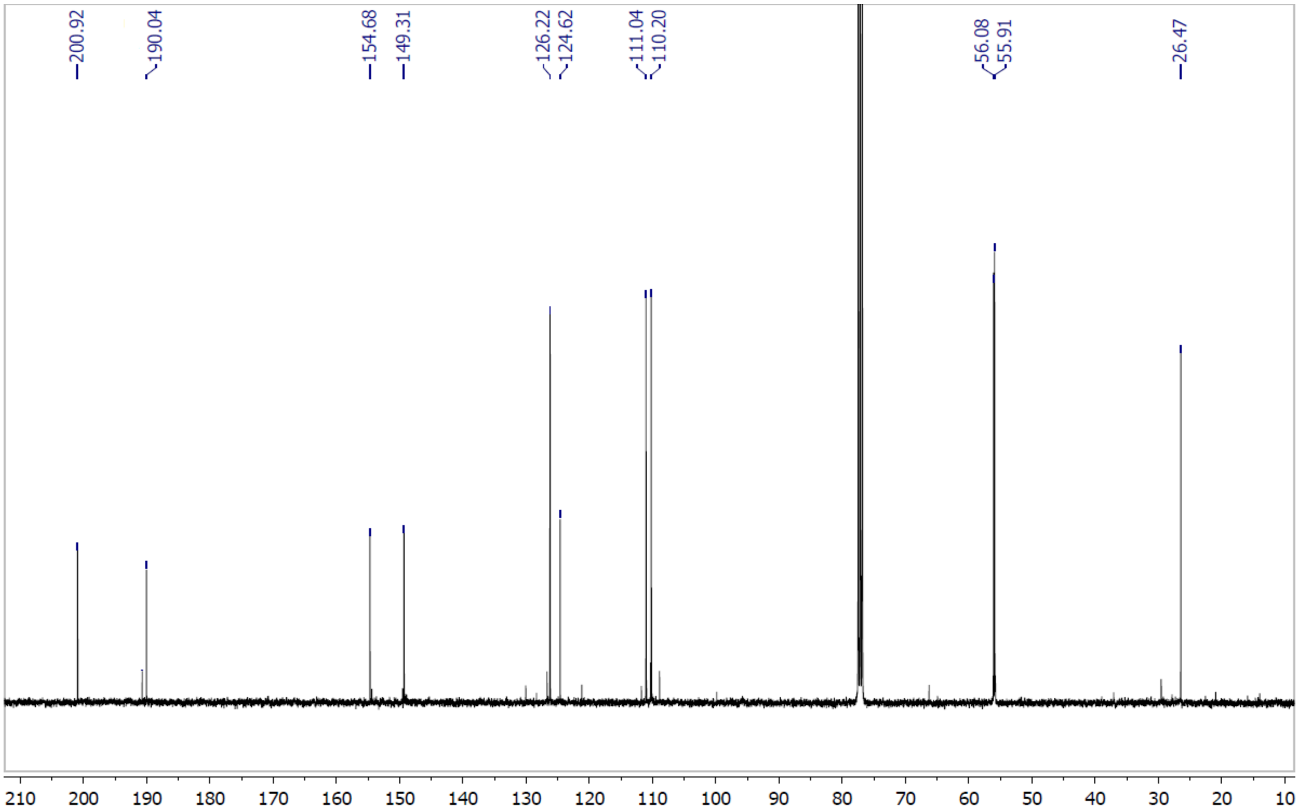

Figure S24. ^{13}C NMR spectrum of 1-(3,4-dimethoxyphenyl)propane-1,2-dione.

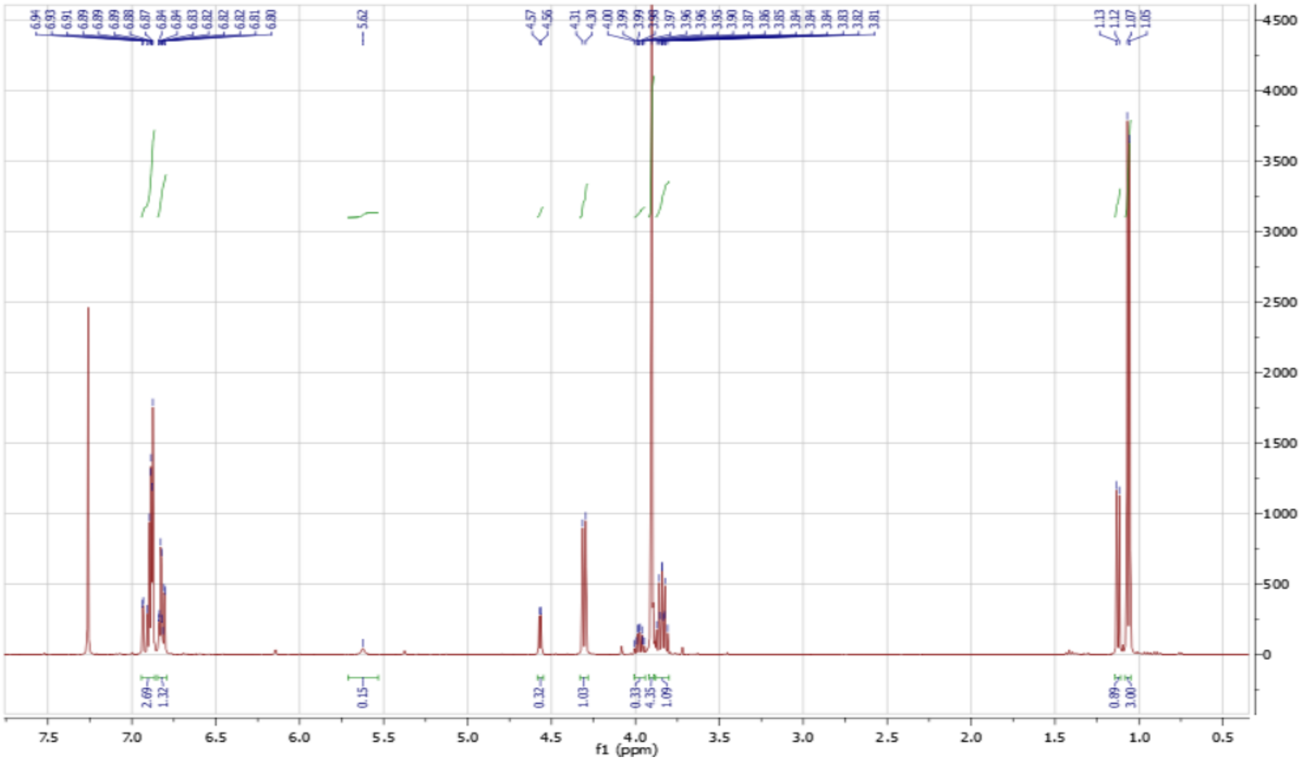

Figure S25. ^{1}H NMR spectrum of (1*R**,2*S**) and (1*R**,2*R**)-1-(4-hydroxy-3-methoxyphenyl)propane-1,2-diol (**5b**).

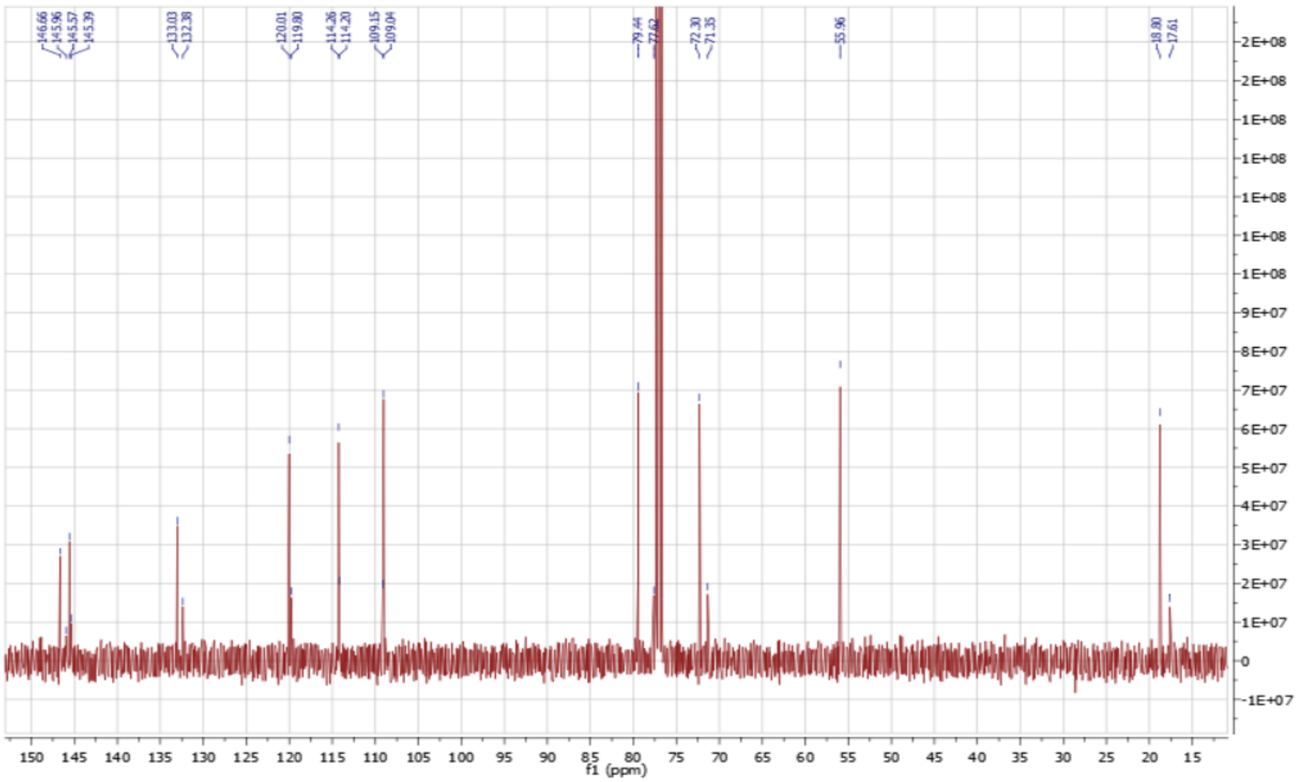

Figure S26. ^{13}C NMR spectrum of (1*R**,2*S**) and (1*R**,2*R**)-1-(4-hydroxy-3-methoxyphenyl)propane-1,2-diol (**5b**).

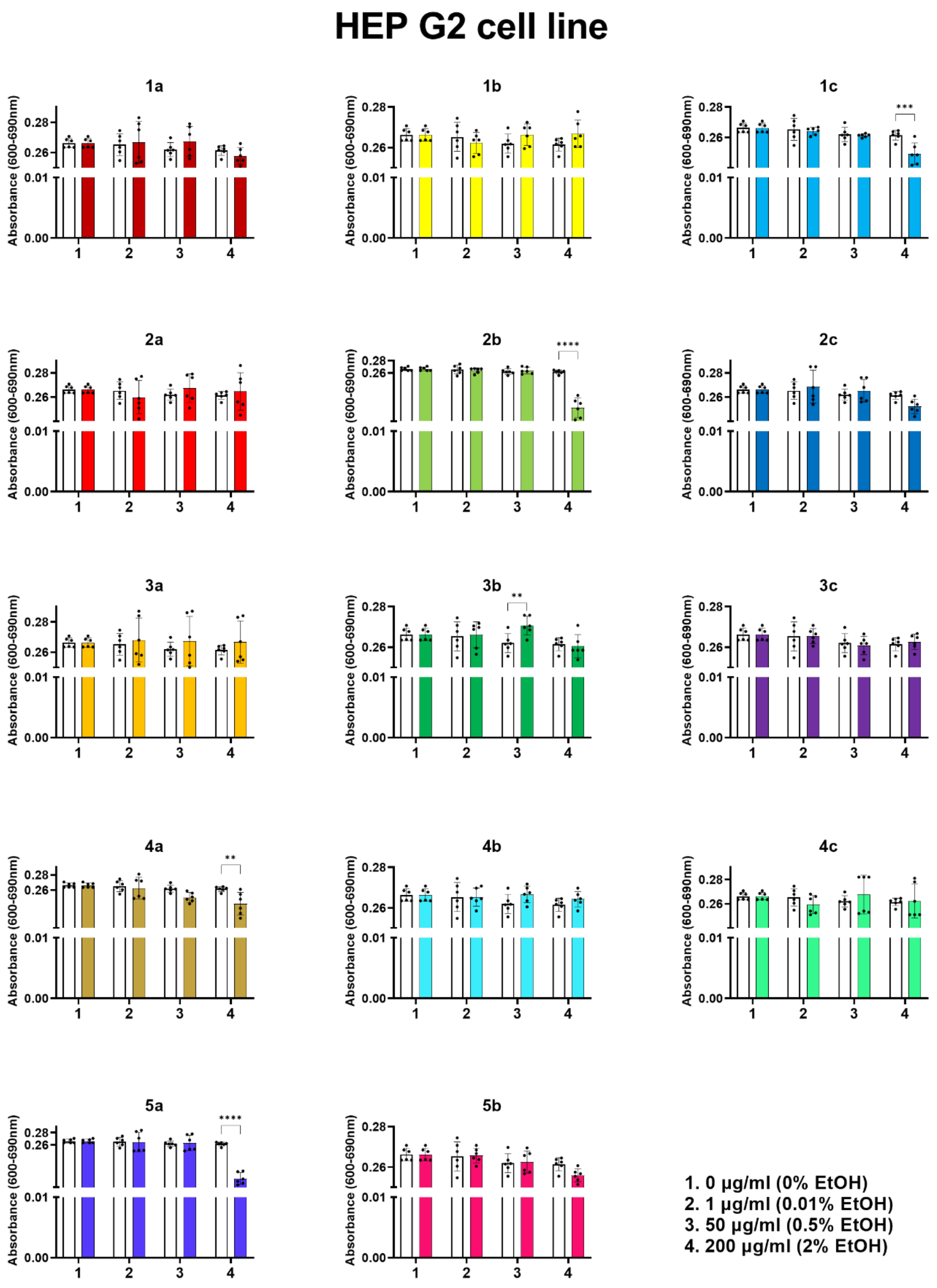

Figure S27. Absorbance values of HepG2 cell line treated with the compounds at concentrations from 0 to 200 µg/ml. The results are shown as mean values ± standard deviations.

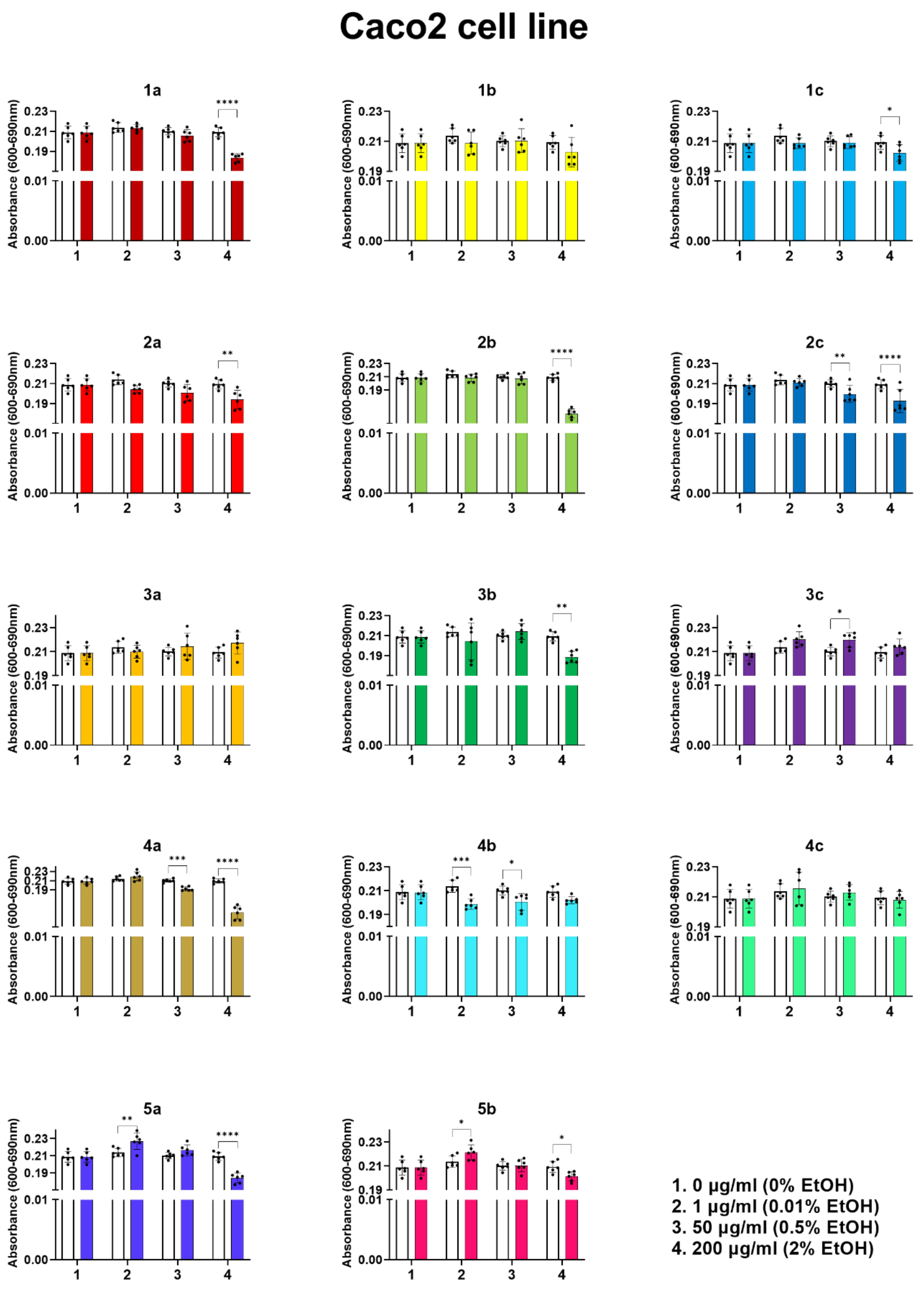

Figure S28. Absorbance values of Caco-2 cell line treated with the compounds at concentrations from 0 to 200 µg/ml. The results are shown as mean values ± standard deviations.

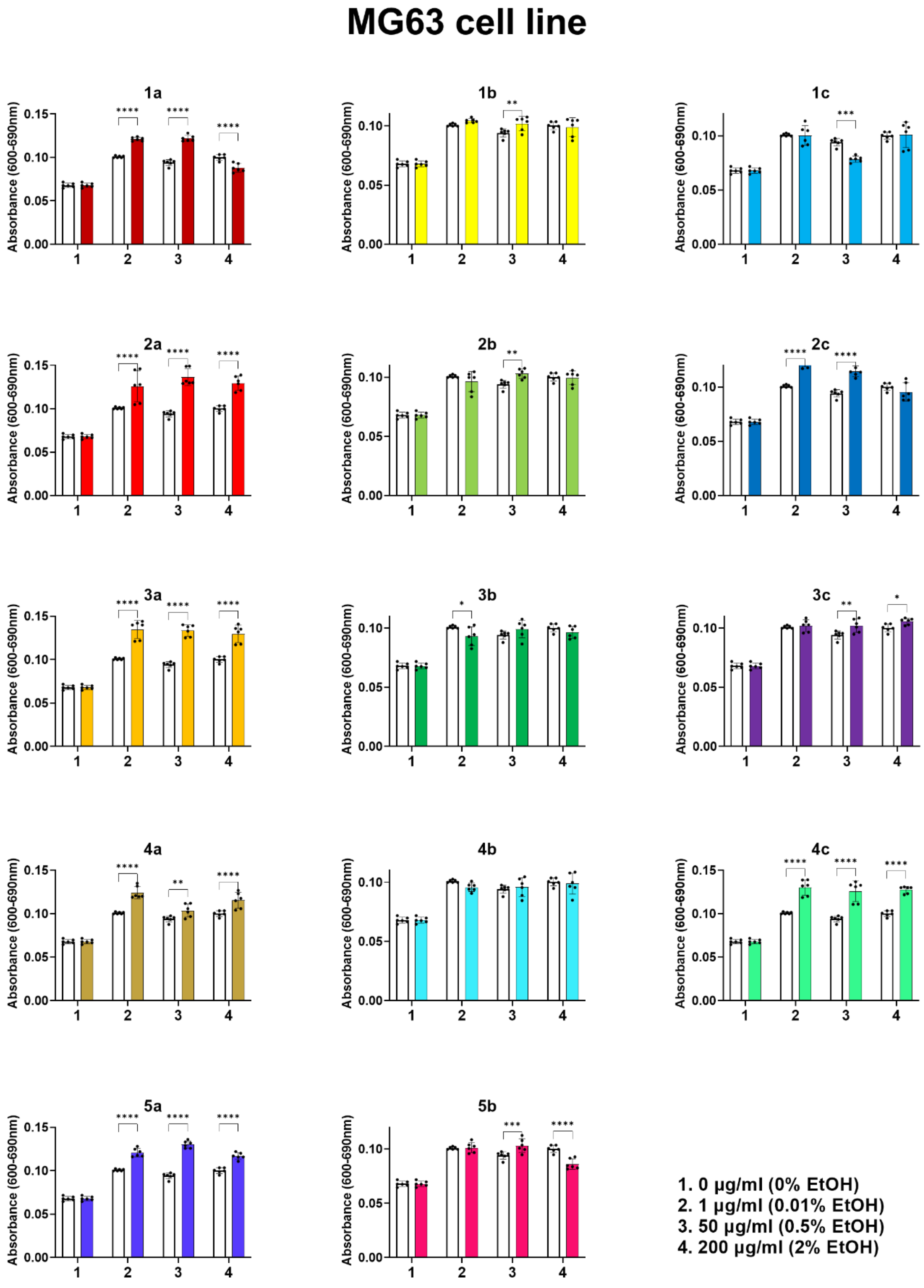

Figure S29. Absorbance values of MG63 cell line treated with the compounds at concentrations from 0 to 200 µg/ml. The results are shown as mean values ± standard deviations.

Table S1. Results of the screening-scale biotransformations of diol **1b**.

Strain	Time [days]	Substrate conversion	Chemical composition of biotransformation products		
		1-(1,3-benzodioxol-5-yl)propane-1,2-diol **1b [%]**	1-(1,3-benzodioxol-5-yl)propane-1,2-dione **[%]**	1-(1,3-benzodioxol-5-yl)-1-hydroxypropan-2-one **[%]**	1-(1,3-benzodioxol-5-yl)-2-hydroxypropan-1-one **1c [%]**
Bacillus subtilis PCM2238	3	44 (±2.1)	8 (±0.7)	10 (±1.7)	26 (±1.1)
	7	47 (±3.1)	4 (±0.5)	10 (±1.9)	33 (±3.2)
	11	58 (±3.4)	4 (±0.5)	10 (±0.9)	44 (±3.5)
Bacillus subtilis PCM2850	3	28 (±1.5)	7 (±0.7)	8 (±0.5)	13 (±0.9)
	7	26 (±2.3)	5 (±0.3)	10 (±0.9)	21 (±2.1)
	11	58 (±2.9)	5 (±0.3)	10 (±1.1)	43 (±2.3)
Dietzia maris PCM2292	3	0	-	-	-
	7	0	-	-	-
	11	0	-	-	-
Dietzia sp. DSM44016	3	66 (±2.4)	6 (±0.2)	12 (±0.5)	48 (±2.1)
	7	84 (±3.1)	6 (±0.2)	12 (±1.0)	66 (±3.3)
	11	100	5 (±0.4)	10 (±0.9)	85 (±3.9)
Gordonia bronchialis PCM2167	3	21 (±1.0)	2 (±0.1)	5 (±0.1)	14 (±1.2)
	7	21 (±1.6)	4 (±0.8)	5 (±0.5)	12 (±1.1)
	11	63 (±2.0)	13 (±1.7)	17 (±0.9)	33 (±2.3)
Gordonia rubripertincta PCM2144	3	40 (±2.5)	2 (±0.2)	6 (±0.8)	32 (±2.1)
	7	53 (±3.1)	4 (±0.3)	11 (±1.4)	38 (±1.7)
	11	65 (±4.1)	7 (±0.6)	12 (±1.1)	46 (±3.4)
Micrococcus luteus PCM525	3	13 (±0.7)	-	-	13 (±0.7)
	7	10 (±1.3)	-	-	10 (±1.3)
	11	17 (±1.9)	-	-	17 (±1.9)
Pseudomonas aeruginosa PCM2720	3	0	-	-	-
	7	0	-	-	-
	1	0	-	-	-
Pseudomonas aeruginosa PCM3035	3	0	-	-	-
	7	0	-	-	-
	11	0	-	-	-
Rhodococcus coprophilus PCM2174	3	0	-	-	-
	7	15 (±1.3)	3 (±0.1)	-	12 (±1.3)
	11	35 (±3.4)	2 (±0.1)	4 (±0.5)	29 (±1.9)
	3	45 (±4.2)	5 (±0.1)	10 (±1.0)	30 (±3.5)

Rhodococcus erythropolis PCM2150	7	61 (±3.1)	4 (±0.3)	11 (±0.7)	46 (±4.2)
	11	79 (±3.5)	4 (±0.6)	12 (±1.0)	63 (±2.9)
Rhodococcus erythropolis DSM44534	3	62 (±3.4)	4 (±0.3)	5 (±0.3)	53 (±3.3)
	7	83 (±2.3)	3 (±0.5)	8 (±0.6)	72 (±1.3)
	11	84 (±3.0)	3 (±0.1)	8 (±0.9)	73 (±3.6)
Rhodococcus rhodnii PCM2157	3	15 (±1.0)	-	5 (±0.6)	10 (±0.3)
	7	40 (±1.9)	2 (±0.1)	5 (±0.2)	33 (±1.6)
	11	49 (±2.8)	2 (±1.0)	4 (±0.1)	43 (±2.4)
Rhodococcus rhodochrous PCM909	3	30 (±1.5)	-	-	30 (±1.5)
	7	42 (±2.3)	-	-	42 (±2.3)
	11	47 (±2.7)	-	-	47 (±2.7)
Rhodococcus ruber PCM2166	3	61 (±3.7)	11 (±0.3)	10 (±1.2)	40 (±3.1)
	7	65 (±4.1)	10 (±1.2)	10 (±0.9)	45 (±4.2)
	11	95 (±3.7)	15 (±0.4)	18 (±1.1)	62 (±2.6)
Rhodococcus ruber PCM2171	3	40 (±2.1)	-	-	40 (±2.1)
	7	44 (±2.5)	2 (±0.1)	5 (±0.3)	37 (±1.9)
	11	49 (±3.5)	2 (±0.1)	5 (±0.5)	42 (±3.1
Rhodococcus ruber PCM2216	3	31 (±2.1)	-	-	31 (±2.1)
	7	40 (±2.1)	4 (±1.4)	2 (±0.1)	34 (±1.6)
	11	54 (±3.5)	5 (±1.0)	6 (±0.4)	43 (±3.3)
Serratia liquefaciens PCM2830	3	0	-	-	-
	7	0	-	-	-
	11	0	-	-	-
Serratia marcescens PCM549	3	0	-	-	-
	7	0	-	-	-
	11	0	-	-	-
Serratia plumuthica PCM550	3	0	-	-	-
	7	0	-	-	-
	11	0	-	-	-
Serratia sp. PCM1324	3	0	-	-	-
	7	0	-	-	-
	11	0	-	-	-
Streptomyces griseus subsp. *Griseus* PCM2331	3	0	-	-	-
	7	0	-	-	-
	11	0	-	-	-

%, determined by GC

Table S2. Results of the screening-scale biotransformations of diol **2b**.

**Strain**	**Time [days]**	**Substrate Conversion** 1-phenylpropane-1,2-diol **2b [%]**	**Chemical composition of biotransformation products** 1-phenylpropane-1,2-dione **[%]**	1-hydroxy-1-phenylpropan-2-one **[%]**	2-hydroxy-1-phenylpropan-1-one **2c [%]**
Bacillus subtilis PCM2238	3	10 (±0.9)	-	-	10 (±0.9)
	7	14 (±1.7)	-	-	14 (±1.7)
	11	29 (±3.1)	4 (±0.2)	5 (±0.6)	20 (±2.5)
Bacillus subtilis PCM2850	3	17 (±2.3)	-	-	17 (±2.3)
	7	20 (±1.8)	-	3 (±0.3)	17 (±1.5)
	11	26 (±3.3)	-	6 (±0.2)	20 (±3.1)
Dietzia maris PCM2292	3	0	-	-	-
	7	0	-	-	-
	11	0	-	-	-
Dietzia sp. DSM44016	3	57 (±4.1)	2 (±0.1)	10 (±1.3)	45 (±4.1)
	7	84 (±3.7)	3 (±0.1)	11 (±1.7)	70 (±2.9)
	11	88 (±3.4)	3 (±0.1)	11 (±1.6)	74 (±3.4)
Gordonia bronchialis PCM2167	3	52 (±1.8)	-	7 (±0.5)	45 (±1.3)
	7	63 (±2.2)	-	8 (±0.7)	55 (±1.7)
	11	67 (±2.7)	-	8 (±0.4)	59 (±2.3)
Gordonia rubripertincta PCM2144	3	37 (±2.7)	3 (±0.1)	7 (±0.3)	27 (±2.3)
	7	35 (±3.3)	-	5 (±0.2)	30 (±3.1)
	11	41 (±2.6)	2 (±0.1)	7 (±0.4)	32 (±1.9)
Micrococcus luteus PCM525	3	0	-	-	-
	7	9 (±0.4)	-	2 (±0.1)	7 (±0.3)
	11	23 (±1.6)	-	6 (±0.2)	17 (±1.4)
Pseudomonas aeruginosa PCM2720	3	0	-	-	-
	7	22 (±2.0)	-	7 (±0.4)	15 (±1.6)
	1	58 (±3.1)	-	13 (±0.7)	45 (±2.4)
Pseudomonas aeruginosa PCM3035	3	0	-	-	-
	7	0	-	-	-
	11	0	-	-	-
Rhodococcus coprophilus PCM2174	3	0	-	-	-
	7	0	-	-	-
	11	0	-	-	-
	3	19 (±1.0)	-	-	19 (±1.0)

Rhodococcus erythropolis PCM2150	7	52 (±2.7)	-	6 (±0.3)	46 (±2.4)
	11	84 (±5.0)	3	14 (±0.9)	67 (±4.1)
Rhodococcus erythropolis DSM44534	3	27 (±2.0)	-	3 (±0.1)	24 (±1.9)
	7	57 (±3.6)	-	5 (±0.2)	52 (±3.4)
	11	80 (±2.5)	5 (±0.2)	5 (±0.2)	70 (±2.1)
Rhodococcus rhodnii PCM2157	3	11 (±1.0)	-	5 (±0.3)	11 (±0.7)
	7	24 (±1.4)	-	5 (±0.2)	19 (±1.2)
	11	37 (±1.9)	-	9 (±0.5)	28 (±1.4)
Rhodococcus rhodochrous PCM909	3	7 (±0.5)	-	-	7 (±0.5)
	7	28 (±2.1)	-	7 (±0.6)	21 (±1.5)
	11	40 (±2.4)	-	6 (±0.3)	34 (±2.1)
Rhodococcus ruber PCM2166	3	38 (±2.9)	6 (±0.2)	8 (±0.6)	24 (±2.1)
	7	61 (±3.7)	3 (±0.1)	10 (±0.8)	48 (±2.8)
	11	96 (±3.8)	-	9 (±0.5)	87 (±3.3)
Rhodococcus ruber PCM2171	3	13 (±1.2)	-	5 (±0.7)	8 (±0.5)
	7	32 (±2.9)	-	14 (±1.8)	18 (±1.1)
	11	41 (±3.3)	-	14 (±1.3)	27 (±2.0)
Rhodococcus ruber PCM2216	3	17 (±0.9)	-	-	17 (±0.9)
	7	50 (±3.3)	5 (±0.1)	9 (±0.3)	36 (±2.9)
	11	52 (±4.1)	4 (±0.3)	13 (±0.7)	35 (±3.1)
Serratia liquefaciens PCM2830	3	25 (±1.7)	-	10 (±0.4)	15 (±1.3)
	7	28 (±2.5)	-	10 (±0.6)	18 (±1.9)
	11	46 (±3.8)	2 (±0.3)	11 (±1.2)	33 (±2.3)
Serratia marcescens PCM549	3	0	-	-	-
	7	20 (±2.0)	-	-	20 (±2.0)
	11	27 (±2.1)	-	4 (±0.2)	23 (±1.9)
Serratia plumuthica PCM550	3	0	-	-	-
	7	0	-	-	-
	11	0	-	-	-
Serratia sp. PCM1324	3	27 (±3.1)	-	8 (±0.9)	19 (±2.2)
	7	47 (±3.8)	-	11 (±0.4)	36 (±3.4)
	11	58 (±3.8)	4 (±0.2)	14 (±1.1)	40 (±2.5)
Streptomyces griseus subsp. *Griseus* PCM2331	3	0	-	-	-
	7	0	-	-	-
	11	0	-	-	-

%, determined by GC

Table S3. Results of the screening-scale biotransformations of diol **3b**.

		**Substrate Conversion**	**Chemical composition of biotransformation products**		
**Strain**	**Time [days]**	1-(4-methoxyphenyl)propane-1,2-diol **3b [%]**	1-(4-methoxyphenyl)propane-1,2-dione **[%]**	1-hydroxy-1-(4-methoxyphenyl)propan-2-one **[%]**	2-hydroxy-1-(4-methoxyphenyl)propan-1-one **3c [%]**
Bacillus subtilis PCM2238	3	0	-	-	-
	7	0	-	-	-
	11	0	-	-	-
Bacillus subtilis PCM2850	3	26 (±2.2)	-	-	26 (±2.2)
	7	36 (±2.4)	-	-	36 (±2.4)
	11	41 (±3.1)	-	-	41 (±3.1)
Dietzia maris PCM2292	3	0	-	-	-
	7	0	-	-	-
	11	0	-	-	-
Dietzia sp. DSM44016	3	16 (±1.6)	-	4 (±0.1)	12 (±1.5)
	7	43 (±2.1)	1 (±0.1)	1 (±0.1)	41 (±1.9)
	11	88 (±5.1)	3 (±0.1)	11 (±0.7)	74 (±4.3)
Gordonia bronchialis PCM2167	3	0	-	-	-
	7	5 (±0.2)	-	1 (±0.1)	4 (±0.1)
	11	30 (±1.5)	2 (±0.1)	3 (±0.1)	25 (±1.3)
Gordonia rubripertincta PCM2144	3	0	-	-	-
	7	0	-	-	-
	11	0	-	-	-
Micrococcus luteus PCM525	3	11 (±0.6)	-	4 (±0.1)	7 (±0.5)
	7	29 (±2.2)	1 (±0.1)	4 (±0.2)	24 (±1.9)
	11	43 (±2.0)	2 (±0.1)	5 (±0.2)	36 (±1.7)
Pseudomonas aeruginosa PCM2720	3	0	-	-	-
	7	0	-	-	-
	1	0	-	-	-
Pseudomonas aeruginosa PCM3035	3	0	-	-	-
	7	0	-	-	-
	11	0	-	-	-
Rhodococcus coprophilus PCM2174	3	0	-	-	-
	7	0	-	-	-
	11	0	-	-	-
	3	21 (±2.8)	-	3 (±0.2)	18 (±2.6)

Rhodococcus erythropolis	7	30 (±3.2)	-	3 (±0.1)	27 (±3.1)
PCM2150	11	42 (±3.2)	2 (±0.1)	2 (±0.1)	38 (±3.0)
Rhodococcus erythropolis	3	29 (±3.3)	-	2 (±0.1)	27 (±3.2)
	7	63 (±3.1)	-	2 (±0.1)	61 (±3.0)
DSM44534	11	90 (±2.5)	1 (±0.1)	7 (±0.4)	82 (±2.0)
Rhodococcus rhodnii	3	0	-	-	-
	7	9 (±0.7)	-	2 (±0.1)	7 (±0.6)
PCM2157	11	21 (±1.8)	-	2 (±0.1)	19 (±1.7)
Rhodococcus rhodochrous	3	10 (±1.1)	-	-	10 (±1.1)
	7	17 (±1.4)	-	2 (±0.1)	15 (±1.3)
PCM909	11	28 (±2.0)	-	2 (±0.1)	26 (±1.9)
Rhodococcus ruber	3	42 (±4.3)	-	4 (±0.1)	38 (±4.2)
	7	78 (±3.6)	-	14 (±0.5)	64 (±3.1)
PCM2166	11	100 (±2.6)	-	12 (±0.5)	88 (±2.1)
Rhodococcus ruber	3	21 (±1.3)	-	7 (±0.4)	14 (±0.9)
	7	37 (±2.0)	3 (±0.1)	10 (±0.8)	23 (±1.1)
PCM2171	11	38 (±2.6)	4 (±0.2)	10 (±0.7)	24 (±1.7)
Rhodococcus ruber	3	28 (±1.8)	-	-	28 (±1.8)
	7	59 (±3.5)	3 (±0.1)	13 (±1.0)	43 (±2.4)
PCM2216	11	59 (±2.9)	2 (±0.1)	10 (±0.7)	47 (±2.1)
Serratia liquefaciens	3	28 (±1.4)	-	12 (±0.3)	16 (±1.1)
	7	41 (±3.2)	-	10 (±0.9)	31 (±2.3)
PCM2830	11	56 (±3.8)	4 (±0.1)	13 (±0.4)	39 (±3.3)
Serratia marcescens	3	13 (±0.5)	-	8 (±0.4)	5 (±0.1)
	7	50 (±2.6)	-	9 (±0.5)	41 (±2.1)
PCM549	11	63 (±3.6)	6 (±0.1)	14 (±1.1)	43 (±2.4)
Serratia plumuthica	3	0	-	-	-
	7	0	-	-	-
PCM550	11	0	-	-	-
Serratia sp.	3	26 (±1.4)	-	5 (±0.1)	21 (±1.3)
PCM1324	7	37 (±1.5)	-	10 (±0.3)	27 (±1.2)
	11	53 (±3.3)	-	11 (±0.9)	42 (±2.4)
Streptomyces griseus subsp. *Griseus*	3	0	-	-	-
	7	0	-	-	-
PCM2331	11	0	-	-	-

%, determined by GC

Table S4. Results of the screening-scale biotransformations of diol **4b**.

Strain	Time [days]	Substrate Conversion 1-(3,4-dimethoxyphenyl)propane-1,2-diol **4b [%]**	Chemical composition of biotransformation products 1-(3,4-dimethoxyphenyl)propane-1,2-dione **[%]**	1-(3,4-dimethoxyphenyl)-1-hydroxypropan-2-one **[%]**	1-(3,4-dimethoxyphenyl)-2-hydroxypropan-1-one **4c [%]**
Bacillus subtilis PCM2238	3	7 (±0.6)	-	-	7 (±0.6)
	7	23 (±1.3)	3 (±0.1)	4 (±0.1)	16 (±1.1)
	11	34 (±2.0)	3 (±0.1)	7 (±0.3)	24 (±1.6)
Bacillus subtilis PCM2850	3	16 (±1.2)	-	2 (±0.1)	14 (±1.1)
	7	26 (±1.8)	-	3 (±0.1)	23 (±1.7)
	11	37 (±1.2)	-	6 (±0.2)	31 (±1.0)
Dietzia maris PCM2292	3	0	-	-	-
	7	0	-	-	-
	11	0	-	-	-
Dietzia sp. DSM44016	3	47 (±3.9)	-	8 (±0.3)	39 (±3.6)
	7	70 (±5.1)	4 (±0.1)	8 (±0.3)	58 (±4.7)
	11	89 (±2.7)	7 (±0.1)	13 (±0.7)	69 (±1.9)
Gordonia bronchialis PCM2167	3	42 (±1.1)	-	5 (±0.1)	37 (±1.1)
	7	63 (±2.1)	-	5 (±0.1)	58 (±2.0)
	11	76 (±2.3)	-	6 (±0.5)	70 (±1.8)
Gordonia rubripertincta PCM2144	3	25 (±2.0)	-	8 (±0.3)	17 (±1.7)
	7	47 (±2.7)	4 (±0.1)	8 (±0.3)	35 (±2.3)
	11	54 (±2.5)	4 (±0.1)	7 (±0.6)	43 (±1.8)
Micrococcus luteus PCM525	3	7 (±0.9)	-	-	7 (±0.9)
	7	20 (±1.6)	-	7 (±0.1)	13 (±1.5)
	11	29 (±3.0)	-	7 (±1.1)	22 (±1.9)
Pseudomonas aeruginosa PCM2720	3	0	-	-	-
	7	0	-	-	-
	1	0	-	-	-
Pseudomonas aeruginosa PCM3035	3	0	-	-	-
	7	0	-	-	-
	11	0	-	-	-
Rhodococcus coprophilus PCM2174	3	0	-	-	-
	7	0	-	-	-
	11	0	-	-	-
	3	25 (±3.3)	-	-	25 (±3.3)

Rhodococcus erythropolis PCM2150	7	67 (±5.3)	4 (±0.1)	12 (±1.0)	51 (±4.2)
	11	86 (±2.9)	2 (±0.1)	13 (±0.7)	71 (±2.1)
Rhodococcus erythropolis DSM44534	3	31 (±1.8)	-	4 (±0.1)	27 (±1.7)
	7	63 (±2.9)	5 (±0.1)	6 (±0.4)	52 (±2.4)
	11	94 (±4.6)	7 (±0.8)	8 (±0.9)	79 (±2.9)
Rhodococcus rhodnii PCM2157	3	21 (±2.6)	-	6 (±0.5)	15 (±2.1)
	7	23 (±2.5)	-	6 (±0.5)	17 (±2.0)
	11	31 (±3.6)	-	7 (±1.1)	24 (±2.5)
Rhodococcus rhodochrous PCM909	3	25 (±3.2)	-	-	25 (±3.2)
	7	41 (±4.7)	-	10 (±1.0)	31 (±3.7)
	11	50 (±3.1)	-	11 (±0.8)	39 (±2.3)
Rhodococcus ruber PCM2166	3	44 (±2.9)	-	10 (±1.1)	34 (±1.8)
	7	70 (±3.3)	6 (±0.1)	13 (±0.7)	51 (±2.5)
	11	98 (±2.0)	8 (±0.5)	13 (±1.0)	77 (±2.0)
Rhodococcus ruber PCM2171	3	15 (±0.9)	-	4 (±0.1)	11 (±0.8)
	7	35 (±2.4)	-	13 (±0.6)	22 (±1.8)
	11	41 (±3.6)	-	12 (±1.5)	29 (±2.1)
Rhodococcus ruber PCM2216	3	20 (±1.8)	-	-	20 (±1.8)
	7	24 (±1.8)	-	-	24 (±1.8)
	11	25 (±2.5)	-	-	25 (±2.5)
Serratia liquefaciens PCM2830	3	25 (±2.3)	-	10 (±0.8)	15 (±1.5)
	7	30 (±3.9)	-	13 (±1.5)	17 (±2.4)
	11	54 (±4.0)	5 (±0.3)	14 (±0.8)	35 (±2.9)
Serratia marcescens PCM549	3	7 (±0.9)	-	-	7 (±0.9)
	7	28 (±2.5)	-	5 (±0.6)	23 (±1.9)
	11	33 (±2.6)	-	6 (±0.3)	27 (±2.3)
Serratia plumuthica PCM550	3	0	-	-	-
	7	0	-	-	-
	11	0	-	-	-
Serratia sp. PCM1324	3	21 (±1.7)	-	7 (±0.3)	14 (±1.4)
	7	45 (±3.2)	-	12 (±0.7)	33 (±2.5)
	11	59 (±3.5)	8 (±0.4)	12 (±0.9)	39 (±2.2)
Streptomyces griseus subsp. *Griseus* PCM2331	3	0	-	-	-
	7	0	-	-	-
	11	0	-	-	-

%, determined by GC

Table S5. Percentage of haemolysis of human RBCs after a 1h incubation with the compounds at various concentrations. The results are shown as mean values ± standard deviations.

**Compound**	**Concentration [µM]**						
	**0**	**10**	**20**	**40**	**60**	**80**	**100**
**Control**	1.340 ± 0.416	1.583 ± 0.202	1.741 ± 0.007	1.820 ± 0.188	2.017 ± 0.091	1.756 ± 0.363	2.146 ± 0.105
**1a**	1.340 ± 0.416	2.412 ± 0.481	1.544 ± 0.202	2.180 ± 0.153	2.397 ± 0.446	2.585 ± 0.014	2.550 ± 0.007
**1b**	1.340 ± 0.416	2.279 ± 0.251	1.864 ± 0.293	1.978 ± 0.272	1.884 ± 0.251	2.387 ± 0.349	2.575 ± 0.321
**1c**	1.340 ± 0.416	1.460 ± 0.080	1.171 ± 0.020	1.806 ± 0.302	1.848 ± 0.027	1.412 ± 0.295	1.507 ± 0.576
**2a**	1.340 ± 0.416	1.273 ± 0.319	1.665 ± 0.175	1.475 ± 0.034	1.767 ± 0.155	1.250 ± 0.263	2.042 ± 0.067
**2b**	1.340 ± 0.416	1.038 ± 0.275	1.081 ± 0.107	1.005 ± 0.295	1.317 ± 0.255	1.787 ± 0.382	1.706 ± 0.054
**2c**	1.340 ± 0.416	1.156 ± 0.027	1.858 ± 0.697	1.422 ± 0.550	1.744 ± 0.590	1.938 ± 0.154	2.464 ± 0.322
**3a**	1.340 ± 0.416	1.362 ± 0.296	1.143 ± 0.159	2.235 ± 0.130	2.536 ± 0.267	2.199 ± 0.108	2.500 ± 0.390
**3b**	1.340 ± 0.416	2.451 ± 0.509	2.979 ± 0.153	1.904 ± 0.209	2.294 ± 0.635	2.397 ± 0.209	2.604 ± 0.265
**3c**	1.340 ± 0.416	1.834 ± 0.355	1.649 ± 0.509	1.725 ± 0.174	2.327 ± 0.436	2.152 ± 0.161	1.526 ± 0.134
**4a**	1.340 ± 0.416	1.760 ± 0.152	1.480 ± 0.173	1.669 ± 0.195	1.577 ± 0.180	2.475 ± 0.152	1.786 ± 0.159
**4b**	1.340 ± 0.416	1.635 ± 0.127	1.517 ± 0.375	1.417 ± 0.074	1.199 ± 0.395	1.185 ± 0.040	2.057 ± 0.536
**4c**	1.340 ± 0.416	1.483 ± 0.007	1.934 ± 0.228	2.099 ± 0.395	1.768 ± 0.342	2.024 ± 0.047	2.445 ± 0.013
**5a**	1.340 ± 0.416	1.379 ± 0.047	1.161 ± 0.047	1.753 ±0.134	1.881 ± 0.020	2.047± 0.080	2.185± 0.261
**5b**	1.340 ± 0.416	1.270 ± 0.094	1.550 ± 0.101	1.521 ± 0.181	1.621± 0.174	2.583 ± 0.288	1.981± 0.308

Table S6. Fluorescence anisotropy values of the DPH probe in membranes of RBCs treated with the compounds at 37 °C . The results are shown as mean values ± standard deviations.

Compound	Concentration (μM)			
	0	20	60	100
**Control**	0.266 ± 0.003	0.267 ± 0.006	0.269 ± 0.006	0.266 ± 0.005
**1a**	0.266 ± 0.003	0.262 ± 0.016	0.264 ± 0.030	0.237 ± 0.041
**1b**	0.266 ± 0.003	0.247 ± 0.018	0.274 ± 0.036	0.252 ± 0.012
**1c**	0.266 ± 0.003	0.260 ± 0.011	0.267 ± 0.019	0.277 ± 0.078
**2a**	0.266 ± 0.003	0.262 ± 0.020	0.268 ± 0.023	0.267± 0.006
**2b**	0.266 ± 0.003	0.286 ± 0.015	0.277 ± 0.028	0.278 ± 0.011
**2c**	0.266 ± 0.003	0.236 ± 0.026	0.240 ± 0.019	0.238 ± 0.002
**3a**	0.266 ± 0.003	0.263 ± 0.005	0.261 ± 0.009	0.259 ± 0.008
**3b**	0.266 ± 0.003	0.272 ± 0.019	0.249 ± 0.083	0.262 ± 0.056
**3c**	0.266 ± 0.003	0.239 ± 0.006	0.249 ± 0.007	0.244 ± 0.003
**4a**	0.266 ± 0.003	0.264 ± 0.004	0.261 ± 0.008	0.269 ± 0.007
**4b**	0.266 ± 0.003	0.289 ± 0.004	0.228 ± 0.095	0.290 ± 0.003
**4c**	0.266 ± 0.003	0.257 ± 0.014	0.243 ± 0.019	0.263 ± 0.008
**5a**	0.266 ± 0.003	0.259 ± 0.009	0.272 ± 0.085	0.256 ± 0.023
**5b**	0.266 ± 0.003	0.268 ± 0.025	0.277 ± 0.003	0.272 ± 0.058
